# Quercetin boosts gut microbiota-driven production of isovanillic acid to alleviate colitis via enhancing intestinal barrier function

**DOI:** 10.1016/j.crfs.2025.101183

**Published:** 2025-08-23

**Authors:** Liang Lei, Jing Wang, Juanjuan Wang, Wenjuan He, Tao Wu, Jing Li, Xiaobin Bi, Mei Mei, Xinlei Guan, Xiaoqiang Zhu

**Affiliations:** a Central Laboratory, Wuhan Fourth Hospital, Wuhan, China; b Institute of Clinical Pharmacy, Wuhan Fourth Hospital, Wuhan, China; c Department of Gastroenterology, Wuhan Fourth Hospital, Wuhan, China; d Pharmaceutical Department, Hubei Cancer Hospital, Tongji Medical College, Huazhong University of Science and Technology, Wuhan, China; e School of Pharmacy, Tongji Medical College, Huazhong University of Science and Technology, Wuhan, China; f Cancer Center, Union Hospital, Tongji Medical College, Huazhong University of Science and Technology, Wuhan, China

**Keywords:** Quercetin, Ulcerative colitis, Gut barrier function, Intestinal flora, Isovanillic acid (IVA)

## Abstract

Ulcerative colitis (UC) is a severe inflammatory bowel disease marked by intestinal inflammation, compromised barrier function, and gut microbiota imbalance, with a restricted range of therapeutic options currently available. Quercetin, a flavonoid extracted from fruits and vegetables, have been shown significant anti-inflammatory and microbiota-modulating effects. However, the interactions between gut microbes and quercetin in colitis remain insufficiently elucidated. This research delved into the potential involvement of the gut microbiota-isovanillic acid (IVA)-intestinal barrier axis in the anti-colitis effects of quercetin via 16S rDNA sequencing and metabolomics. Quercetin administration effectively enhanced gut barrier integrity and mitigated colitis by boosting IVA production through modulating gut microbiota composition, particularly increasing *Clostridium_XIVa* and *Clostridium_XI* abundances. Notably, gut microbiota depletion with antibiotics (ABX) treatment markedly diminished IVA production and concurrently negated quercetin's positive effects on colitis. More importantly, IVA supplementation was also effective in alleviating colitis via enhancing gut barrier function, suggesting gut microbiota-derived IVA supported beneficial effects of quercetin on colitis. Our research highlighted the crucial involvement of gut microbial-derived IVA in the anti-colitis effects of quercetin and underscored its therapeutic potential, along with IVA, as a treatment for colitis and other intestinal inflammatory conditions.

## Highlights

• Quercetin mitigated colitis via microbiota-isovanillic acid-gut barrier axis.
• Quercetin enriched *Clostridium_XIVa* and *Clostridium_XI* in colitis mice.
• Quercetin promoted isovanillic acid generation through modulating gut microbiota.
• Isovanillic acid, a microbial-derived metabolite, exhibited anticolitic effects.
• Isovanillic acid alleviated intestinal barrier damage *in vivo* and *in vitro.*

## Graphical abstract

**Figure:**
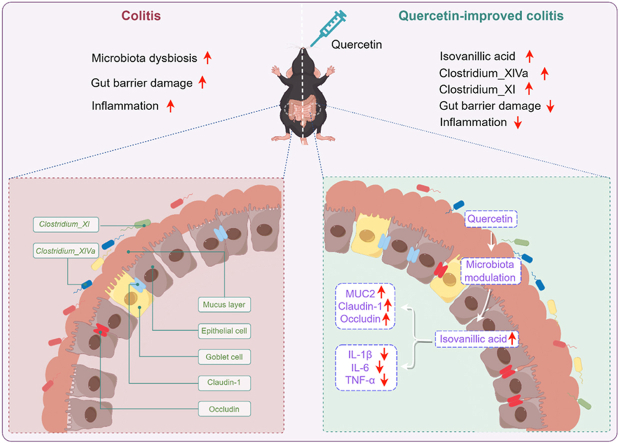

## 1 Introduction

Ulcerative colitis (UC), categorized as an inflammatory bowel disease (IBD), has become a global health challenge. It is estimated that the global prevalence of UC was 5 million cases in 2023 (Le Berre, Honap and Peyrin-Biroulet, 2023). Epidemiological data reveals a pronounced surge in the incidence of UC across low- and middle-income countries over the past decade (Le Berre, Honap and Peyrin-Biroulet, 2023). However, UC continues to present challenges in understanding its etiology and pathogenesis (Kobayashi et al., 2020). Recent research underscores a significant correlation between UC and factors including intestinal inflammation, barrier damage, and microbial dysbiosis (Chen et al., 2021). Despite this, the current landscape of preventive and therapeutic options for UC remains limited (Vieujean et al., 2025). Conventional treatments for UC, such as mesalazine, corticosteroids, and immunosuppressant, are limited by their efficacy, associated side effects, routes of administration, potential risk of neoplasm, and challenges in ensuring patient compliance over long-term use (Kobayashi et al., 2020). Dietary interventions with polyphenols and polysaccharides have emerged as a key strategy for mitigating UC symptoms, demonstrating potential due to their purported capabilities in modifying gut microorganisms, boosting intestinal function, and adjusting immune reactions (Li et al., 2023; Yuan et al., 2023). A thorough elucidation of the protective mechanisms underlying these interventions could lead to improved treatment options for UC.

Quercetin, a flavonoid naturally found in numerous vegetables and fruits, is known for its biological functions, particularly antioxidant, anti-inflammatory, and anti-obesity capabilities (Yan et al., 2023; X. Q. Zhu et al., 2024). Some previous studies have demonstrated the anti-colitis effects of quercetin, and it is believed that protective benefits of quercetin stem from its powerful anti-inflammatory properties, which involves the direct suppression of the NLR family pyrin domain containing 3 (NLRP3) inflammasome (Wu et al., 2024), the nuclear factor kappa B (NF-κB) signaling pathway (Li et al., 2019), the sirtuin 1 (SIRT1)/Akt pathway (Cui et al., 2022), and other anti-inflammatory pathways (Liu et al., 2025). However, the limited oral absorption and bioavailability of quercetin constrains these effects in target tissues (X. A. Zhu et al., 2024). Recent evidences from animal studies suggest that quercetin supplementation may confer benefits for microbial balance, thereby enhancing intestinal barrier function and providing anti-inflammatory benefits (Feng et al., 2023; Zhao et al., 2021). However, the potential mechanisms through which dietary quercetin reduces inflammation and boosts barrier function by interacting with gut microbiota are still unknown. Identifying the precise functions of intestinal bacteria and metabolites modulated by quercetin is of significant importance.

Upon entering the intestine, quercetin is metabolized by the intestinal flora into a series of phenolic acids (X. A. Zhu et al., 2024). Emerging evidence from several studies indicates that specific phenolic acids exhibit remarkable anti-colitis effects (Lu and Han, 2024). For example, vanillic acid (VA) represses ferroptosis and promotes the repair of intestinal barrier damage, thus playing a crucial role in alleviating colitis (Ni et al., 2024; Zhao et al., 2025). Isovanillic acid (IVA), an isomer of VA, has been reported to possess various biological properties, including anti-inflammatory and antioxidant effects (Chen et al., 2023). Nevertheless, whether it shares a similar anti-colitis effect with VA remains an area of uncharted research territory.

This research aimed to explore how gut microbes and their metabolite, IVA, contribute to the beneficial impact of quercetin on dextran sulfate sodium (DSS)-induced colitis via employing 16S rDNA sequencing and untargeted metabolomics. Our findings provided compelling evidence that quercetin-induced remodeling of the gut microorganisms played crucial roles in its effects against colitis. IVA was identified as a significant gut microbiota-derived metabolite involved in this process, which offered substantial protection against colitis via inhibiting inflammation and strengthening gut barrier integrity. These results offered novel insights into the mechanisms by which quercetin ameliorated UC by targeting gut microbiota and IVA production, thereby advancing our understanding of the therapeutic benefits of quercetin and IVA for IBD.

## 2 Materials and methods

### 2.1 Materials

Quercetin with 97 % purity (Yuanye Bio-Technology, China, S25567), carboxymethylcellulose sodium (Aladdin, China, C294622), DSS (40,000 Da; Bidepharm, China, BD123894), vancomycin (Aladdin, China, V301569), metronidazole (Aladdin, China, M109874), neomycin (Aladdin, China, N109017), ampicillin (Aladdin, China, A105483), IVA with 98 % purity (D&B, China, Q107986), hematoxylin and eosin (H&E) dye solution (Servicebio, China, G1005), Alcian blue dye solution (Servicebio, China, G1027), anti-CLDN1 antibody (Servicebio, China, GB111401), anti-OCLN antibody (Servicebio, China, GB152543), RNAiso Plus reagent (Yuanye Bio-Technology, China, R21086), HiScript II Q RT SuperMix for PCR Kit (Vazyme, China, R223), ChamQ Blue Universal SYBR qPCR Master Mix Kit (Vazyme, China, Q312), IL-1β, TNF-α, IL-6, LPS, and MUC2 enzyme-linked immunosorbent assay (ELISA) kits (MSKBIO, China), FITC-labeled dextran (FD4, Beyotime, China, ST2930), Caco-2 cell culture medium (Servicebio, China, GZ100818).

### 2.2 Mice and treatments

Six to eight-week-old male C57BL/6J mice, free of specific pathogens, were sourced from the Hubei Center for Disease Control and Prevention in Wuhan, China. Mice were housed in a controlled environment with a 12-h cycle of light and darkness, temperatures set at 22 ± 2 °C, and humidity at 55 ± 5 %. All animal treatments were conducted in strict compliance with the ethical guidelines set by the Institutional Animal Care and Use Committee of Huazhong University of Science and Technology (IAUCC number: 4485).

Animal experiment 1: To explore the effects of quercetin on colitis, mice were categorized into 3 groups: control group (C, n = 6), colitis model group (M, n = 7) and colitis model + quercetin treatment group (MQ, n = 7) after one-week of adaptive feeding. The MQ mice received a daily administration of 100 mg/kg quercetin dissolved in 0.15 % (w/v) carboxymethylcellulose sodium for two weeks, while other mice were given the same volume of the solvent. After administering quercetin for a week, the M and MQ mice were given water containing 3 % (w/v) DSS, while the C mice kept drinking normal water for one more week. The body weight and disease activity index (DAI) was evaluated as described (Ji et al., 2018).

Animal experiment 2: To investigate the role of the gut microbiota in the amelioration effects of quercetin on colitis, C + ABX, M + ABX, and MQ + ABX mice (n = 8 in each group) were given antibiotics (ABX) mixture, including 0.5 g/L vancomycin, 1 g/L metronidazole, 1 g/L neomycin, and 1 g/L ampicillin, for 2 weeks to deplete gut microbes. After 1 week of ABX treatment, MQ + ABX group were administrated 100 mg/kg quercetin while the C + ABX and M + ABX mice were given same volume of solvent once per day for 2 weeks. The M + ABX and MQ + ABX mice were switched from ABX water to 3 % DSS water in the second week of quercetin treatment, while C + ABX mice were given sterile water.

Animal experiment 3: To further investigate the anti-colitis effects of IVA, the mice were allocated into three groups: a control group (C, n = 7), a colitis model group (M, n = 10), and an IVA-administrated colitis model group (MIVA, n = 10). The MIVA group received an intragastric administration of 100 mg/kg IVA, whereas the C and M mice were given an equal volume of solvent for 2 weeks. After 1 week of IVA treatment, M and MIVA mice were given 3 % DSS drinking water, while C mice continued to drink sterile water for 1 week.

### 2.3 Colon length measurement

At the end of each animal experiment, all the mice were euthanized and the colon segment from the ileocecal region to the rectum was completely dissected. The colon length from the proximal colon to the rectum was accurately measured.

### 2.4 Histology

The distal colon tissues harvested from each group in animal experiment 1, 2, and 3 were fixed in 4 % paraformaldehyde for 12 h, embedded in paraffin, and stained with H&E to assess inflammation, and histological score was determined as described (Yi et al., 2024). Mucus barrier damage was assessed through Alcian blue staining. Immunohistochemical staining was applied to observe colonic expression levels of Claudin-1 and Occludin with anti-CLDN1 antibody (1:500) and anti-OCLN antibody (1:500). Images were captured using a Nikon Eclipse TE2000-U light microscope (NIKON, Japan), and ImageJ software (USA) was employed for statistical analysis. After setting the scale bar, a straight line tool was used to measure the distance from the crypt opening to the base of the crypt as the crypt depth. For each section, 20 crypts were randomly selected for measurement and the average value was calculated with 5 independent slices in each group. The area of immunohistochemical positive regions were quantified as a percentage of positive areas in each image as described (Jensen, 2013).

### 2.5 Quantitative real-time PCR (qRT-PCR)

The RNAiso Plus reagent was employed for colonic RNA extraction from each group in animal experiment 1, 2, and 3, and its concentration was determined using a NanoOne spectrophotomer (Youning Instrument, China). The HiScript II Q RT SuperMix for PCR Kit was adopted to carry out the cDNA synthesis. The qRT-PCR was executed on a 7500 Real Time PCR System (Applied Biosystems, USA) using the ChamQ Blue Universal SYBR qPCR Master Mix Kit. The primer sequences are detailed in Supplementary Table S1.

### 2.6 ELISA

In pre-cooled phosphate-buffered saline (PBS), the weighed colonic tissues from each group in animal experiment 1, 2, and 3 were homogenized using a tissue grinder (Servicebio, China). The supernatant was retrieved post-centrifugation (3000 rpm, 10 min, 4 °C) to assess IL-1β, TNF-α, IL-6, and MUC2 concentrations utilizing ELISA kits as per the guidelines. Serum LPS concentrations were quantified with ELISA kits.

### 2.7 Intestinal permeability test

Following a fasting period of 3 h, the mice from each group in animal experiment 1, 2, and 3 received an oral intake of fluorescein isothiocyanate (FITC)-labeled dextran at a dosage of 500 mg/kg. Serum was collected after 4 h to measure FITC fluorescence intensity and determine the levels of FITC in the serum using the standard curve.

### 2.8 16S rDNA sequencing

Microbial DNA was extracted from fresh fecal samples of each group in animal experiment 1 and 2, then its concentration was measured using a NanoDrop 2000 spectrophotometer (Thermo Scientific, USA). The V4 region of 16S rDNA was amplified and analyzed using the AxyPrep kits and Qubit 2.0 for quantification. The samples were then sequenced on the Illumina MiSeq PE250 for paired-end reads. Default settings were used to filter out low-quality reads, and overlapping paired-end reads were combined into consensus sequences with FLASH (v1.2.11). Using UPARSE, operational taxonomic units (OTUs) were grouped at a 97 % similarity level. The tags for each OTU in each sample were compiled into an abundance table to calculate their relative abundance. Tags were then aggregated into a profiling table to determine the relative abundance of taxa at different taxonomic levels. QIIME 2.0 was used for α and β diversity analyses, while principal component analysis (PCA) and partial least squares discriminant analysis (PLS-DA) were carried out with R 3.1.1. Linear discriminant analysis of effect size (LEfSe) was executed on the ImageGP website (Chen et al., 2024).

### 2.9 Metabolomics

Following gradually dissolving the on ice, 25 mg fecal samples from each group in animal experiment 1 and 2 were precisely weighed, followed by the addition of 800 μL extraction solution (methanol: acetonitrile: water = 2:2:1) and 10 μL of an internal standard. After homogenization, it underwent incubation in a 4 °C water bath for 10 min, followed by a 60-min incubation in a freezer set to −20 °C. The sample underwent centrifugation at 4 °C and 25000 g for 15 min to harvest 600 μL supernatant, which was subsequently desiccated utilizing a freeze vacuum concentrator. Reconstitute the dried sample via adding 600 μL reconstitution solution (methanol: ultrapure water = 9:1). Following another 10-min incubation in a 4 °C water bath, centrifuge again at 4 °C and 25,000 g for 15 min, the resulting supernatant was obtained for LC-MS analysis. A Q Exactive high-resolution mass spectrometer was paired with a Waters UPLC I-Class Plus system for the separation and detection of metabolites Chromatographic and reversed-phase separations were conducted following a previous method (Liu et al., 2017). Samples were collected in both the positive and negative ion modes. A Q Exactive mass spectrometer from Thermo Fisher Scientific, USA, was used to acquire primary and secondary mass spectrometry data. For analysis, mass spectrometry data were brought into Compound Discoverer 3.3 (Thermo Fisher Scientific, USA) and integrated with the BMDB, mzCloud, and ChemSpider databases. This produced a data matrix of metabolite peak areas and identification results for further analysis.

### 2.10 Cells

To study the effects of IVA on the intestinal barrier damage *in vitro*, human Caco-2 cells were maintained in DMEM medium with 20 % fetal bovine serum, 100 μg/mL streptomycin, and 100 U/mL penicillin, at 37 °C in a 5 % CO_2_ incubator. After a 48-h exposure to either 10 μg/mL LPS, 50 μM IVA, or 100 μM IVA, or none, the cells were collected for subsequent qRT-PCR, ELISA, and immunofluorescence analyses.

### 2.11 Immunofluorescence staining

For immunofluorescence, the cell climbing slides treated either 10 μg/mL LPS, 50 μM IVA, or 100 μM IVA, or none for 48 h were rinsed 3 times with PBS for 5 min each, followed by 30-min blocking with 3 % bovine serum albumin at 25 °C. Then the slides were treated with antibodies against Claudin-1 (1:500) and Occludin (1:500) at 4 °C covernight. Following the removal of the primary antibody, the corresponding secondary antibody was added and left to incubate for 50 min at 25 °C. Next, the secondary antibody was washed away and DAPI solution was dripped into slides and stored in darkness at 25 °C for 10 min. The climbing slides were ultimately sealed with anti-fade mounting medium followed by a triple washing procedure. Images were obtained with an Eclipse C1 fluorescence microscope (Nikon, Japan) and mean fluorescence intensity (MFI) was quantified as Integrated fluorescence intensity (IntDen)/Area using ImageJ software (USA).

### 2.12 Statistical analysis

The data are expressed as the mean ± standard error of the mean (SEM). Variables between two groups were compared using a two-tailed Student's t-test, and comparisons among multiple groups were done using a one-way ANOVA with Tukey's post hoc test. The associations between gut microbes and IVA levels were determined with linear regression analysis. Data plotting and analysis were done in GraphPad Prism 8.3.

## 3 Results

### 3.1 Quercetin mitigated the development of colitis

**Fig. 1:**
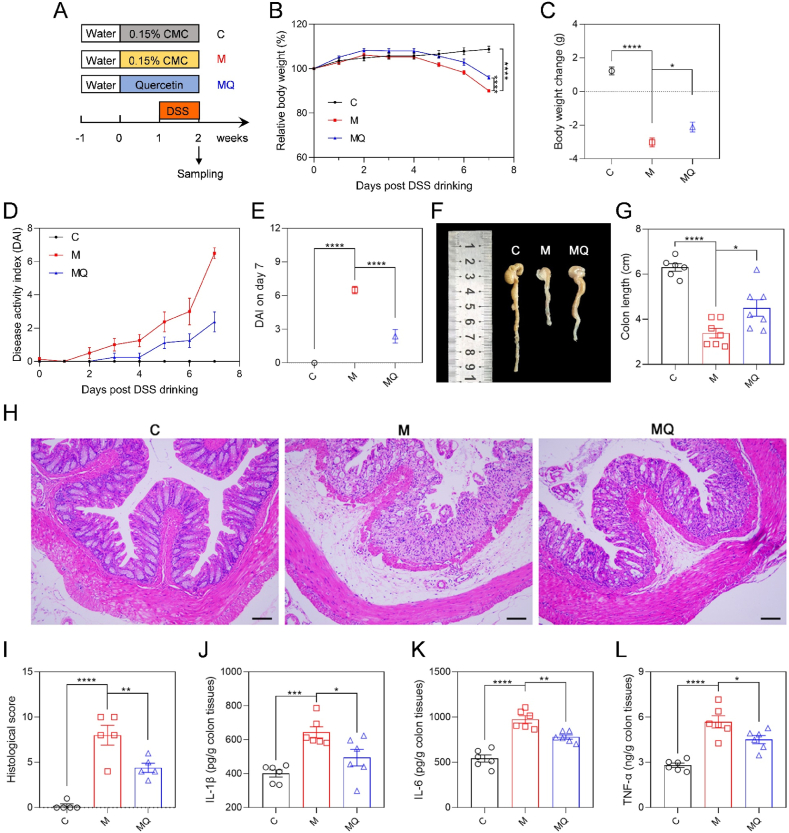
Quercetin effectively improved DSS-induced colitis. (A) Study design of quercetin administration and colitis induction with 3 % DSS. (B) Relative body weight curve. (C) Body weight change in the DSS drinking period. (D, E) DAI curve and DAI on day 7 post DSS drinking. (F, G) Representative images of colon tissues and colon length. (H, I) The H&E staining images of distal colon tissues and histological scores. Scale bar: 100 μm. (J–L) The concentrations of IL-1β, IL-6, and TNF-α in colon tissues determined by ELISA. Data are shown as mean ± SEM, n = 6 in the control (C) group, n = 7 in the colitis model (M) group and quercetin-administrated colitis model (MQ) group in B-G, n = 5 per group in I, n = 6 per group in J-L. The data between M and C or MQ groups was analyzed by one-way ANOVA with Tukey's post hoc test. ∗*p* < 0.05, ∗∗*p* < 0.01, ∗∗∗*p* < 0.001, ∗∗∗∗*p* < 0.0001. Carboxymethylcellulose (CMC); dextran sulfate sodium (DSS); disease activity index (DAI); hematoxylin and eosin (H&E); interleukin 1β (IL-1β); interleukin 6 (IL-6); tumor necrosis factor α (TNF-α).

To study the impact of quercetin on colitis, the mice received an oral dose of quercetin at 100 mg/kg for two weeks, with DSS drinking during the second week of quercetin administration (Fig. 1A). The findings showed that the MQ group of mice exhibited markedly lower body weight loss than the M group (Fig. 1B and C). Notably, the progression of the DAI in the MQ group presented a significantly slower trend compared to the M group (Fig. 1D). Statistical analysis on the 7th day post DSS drinking indicated a substantial 63.4 % decrease in the DAI of colitis mice following quercetin administration (Fig. 1E). This finding was further corroborated by photographic evidence near the anal region of the mice (Fig. S1). Furthermore, quercetin administration obviously mitigated colonic shortening induced by DSS (Fig. 1F and G). The observed reduction in spleen index indicated the anti-inflammatory properties of quercetin (Fig. S2). Histological examination of colon tissues using H&E staining demonstrated that quercetin significantly ameliorated inflammatory cell infiltration, pronounced edema, and crypt loss in mice with colitis (Fig. 1H), which was further confirmed by significantly lower histological scores of the MQ group than the M group (Fig. 1I). Moreover, ELISA analyses demonstrated that quercetin alleviated colonic inflammation by significantly decreased the levels of IL-1β, IL-6, and TNF-α by 23.2 %, 19.9 %, and 20.7 %, respectively, in colitis mice (Fig. 1J–L). And the anti-inflammatory effects of quercetin were also proved by decreased colonic mRNA expression levels of *Il-1β*, *Il-6*, and *Tnf-α* in the MQ group relative to the M group (Fig. S3). The data collectively revealed that quercetin administration effectively mitigated DSS-induced colitis.

### 3.2 Quercetin enhanced gut barrier integrity in colitis

**Fig. 2:**
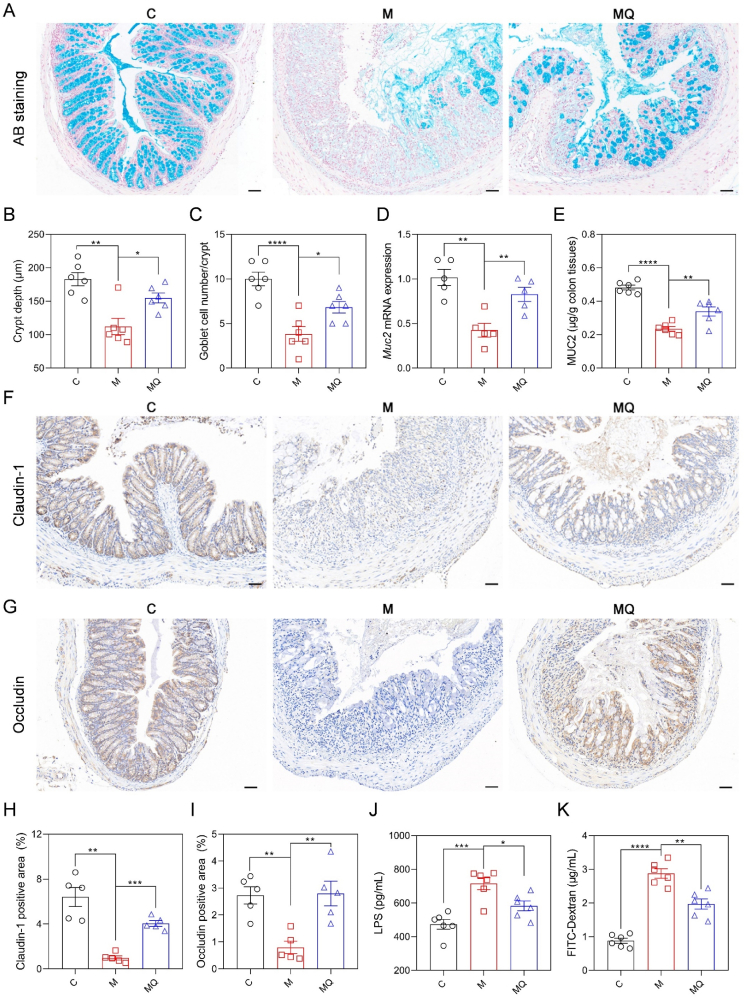
Quercetin protected against gut barrier dysfunction in colitis mice. (A) AB staining images of distal colon tissues. Scale bar: 50 μm. (B) The crypt depth. (C) Goblet cell number per crypt. (D) The qRT-PCR results of *Muc2* mRNA expression levels in colon tissues. (E) ELISA results of the colonic MUC2 content. (F, G) Representative immunohistochemical images of colonic sections stained with Claudin-1 and Occludin antibody. Scale bar: 50 μm. (H, I) The Claudin-1 and Occludin positive area statistics. (J) ELISA results of LPS concentration in serum. (K) Intestinal permeability determined by FITC-dextran concentrations in serum. Data are presented as mean ± SEM, n = 6 in the per group in B-E, J, and K, n = 5 per group in H and I. Comparisons between M and C or MQ groups were analyzed by one-way ANOVA with Tukey's post hoc test. ∗*p* < 0.05, ∗∗*p* < 0.01, ∗∗∗*p* < 0.001, ∗∗∗∗*p* < 0.0001. Alcian blue (AB); mucin-2 (Muc2); lipopolysaccharide (LPS); fluorescein isothiocyanate (FITC).

To explore the involvement of the intestinal mucosal barrier in how quercetin protects against colitis, we conducted Alcian blue (AB) staining on the distal colon tissues and the results indicated that the crypt structure in the M group was gravely‌ compromised, whereas this damage was ameliorated following quercetin administration (Fig. 2A). Statistical analysis revealed crypt depth was reduced by 38.7 % in the M group relative to the C group. Conversely, the MQ group experienced a remarkable 38.2 % elevation in crypt depth compared to the M group (Fig. 2B). Moreover, the quercetin-treated colitis model group exhibited a notable rise in goblet cell count per crypt relative to the untreated colitis model group (Fig. 2C). qRT-PCR analysis showed that quercetin treatment led to a substantial 94.6 % upregulation of *Muc2* mRNA expression in the distal colon tissues (Fig. 2D). Concurrently, ELISA results also indicated that quercetin administration led to a substantial 44.1 % increase in colonic MUC2 protein levels in colitis mice (Fig. 2E). Next, immunohistochemical staining was employed to visualize and quantify the expression of two critical tight junction proteins, Claudin-1 and Occludin, in distal colon tissues. The findings indicated that the positive staining area for Claudin-1 and Occludin of MQ mice was evidently larger than those observed in M mice (Fig. 2F–I). Additionally, qRT-PCR analysis corroborated these results by demonstrating a significant upregulation of *Cldn-1* and *Ocln* mRNA expression levels in MQ mice compared to M mice (Fig. S4). The obvious reduction in serum LPS levels and FITC-Dextran levels observed in the intestinal permeability test in the MQ group compared to the M group further provided robust evidence for quercetin's role in preserving the integrity of the gut barrier (Fig. 2J and K). Together, these results indicated that quercetin significantly reduced gut barrier damage in mice with colitis.

### 3.3 Quercetin reconstructed gut microbial composition in colitis mice

**Fig. 3:**
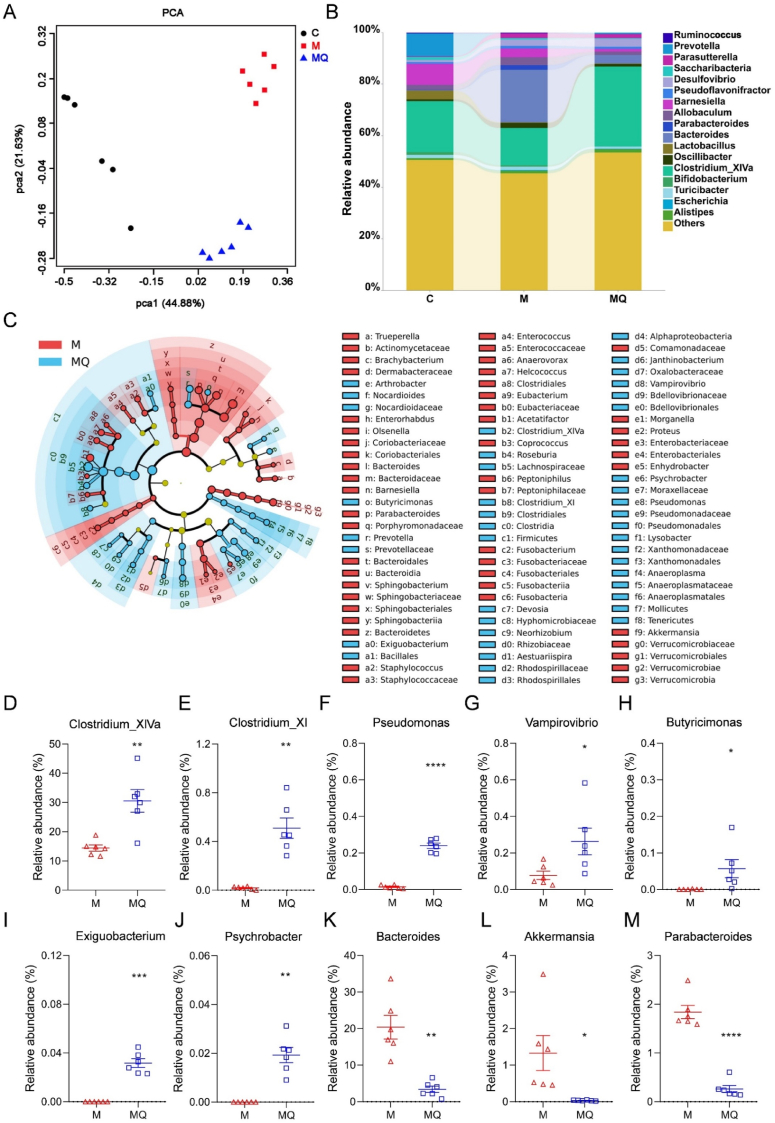
Quercetin ameliorated gut microbiota dysbiosis in mice with colitis. (A) Principal component analysis (PCA). (B) Microbiota structure at the genus level. (C) The linear discriminant analysis of effect size (LEfSe) of gut microbiota between M and MQ groups. (D–M) The relative abundance of *Clostridium_XIVa*, *Clostridium_XI*, *Pseudomonas*, *Vampirovibrio*, *Butyricimonas*, *Exiguobacterium*, *Psychrobacter*, *Bacteroides*, *Akkermansia*, and *Parabacteroides*. Data are presented as mean ± SEM, n = 6 in per group. The data between M and MQ groups was compared using a two-tailed Student's t-test in D-M. ∗*p* < 0.05, ∗∗*p* < 0.01, ∗∗∗*p* < 0.001.

To explore how gut microbiota contributes to quercetin's improvement of colitis, the microbial composition was examined. The α diversity analysis results showed that quercetin did not notably counteract the reduction in α diversity induced by DSS (Fig. S5). However, the PCA, PLS-DA, and enterotype analyses revealed distinctly different clustering patterns between the MQ and M groups (Fig. 3A, S6, S7), suggesting a divergent gut microbiota composition. This divergence was further supported by microbial composition analyses at the levels of both phylum and genus (Figs. S8A and 3B). At the phylum level, quercetin administration resulted in a notable rise in the relative abundance of Firmicutes, along with a reduction in Bacteroidetes and Verrucomicrobia in colitis mice (Fig. S8B). Then LEfSe was employed to further highlight specific differences in bacterial composition among C, M, and MQ groups (Fig. 3C and S9). It was observed that *Clostridium_XIVa*, *Clostridium_XI*, *Pseudomonas*, *Vampirovibrio*, *Butyricimonas*, *Exiguobacterium*, and *Psychrobacter* were significantly enriched by quercetin treatment (Fig. 3D–J). Conversely, the relative levels of *Bacteroides*, *Akkermansia*, and *Parabacteroides* exhibited a notable decline in the MQ group compared to the M group (Fig. 3K–M). Furthermore, quercetin elevated the relative content of *Clostridium_ghonii*, *Clostridium_methylpentosum*, and *Clostridium_xylanolyticum*, while it caused a decrease in *Clostridium_lactatifermentans* and *Clostridium_mangenotii* among *Clostridium* species in colitis mice (Fig. S10). These data provided sufficient evidence that quercetin remodeled the gut microbiota composition and enriched some *Clostridium* species in colitis mice.

### 3.4 The anti-colitis effects of quercetin relied on gut microorganisms

**Fig. 4:**
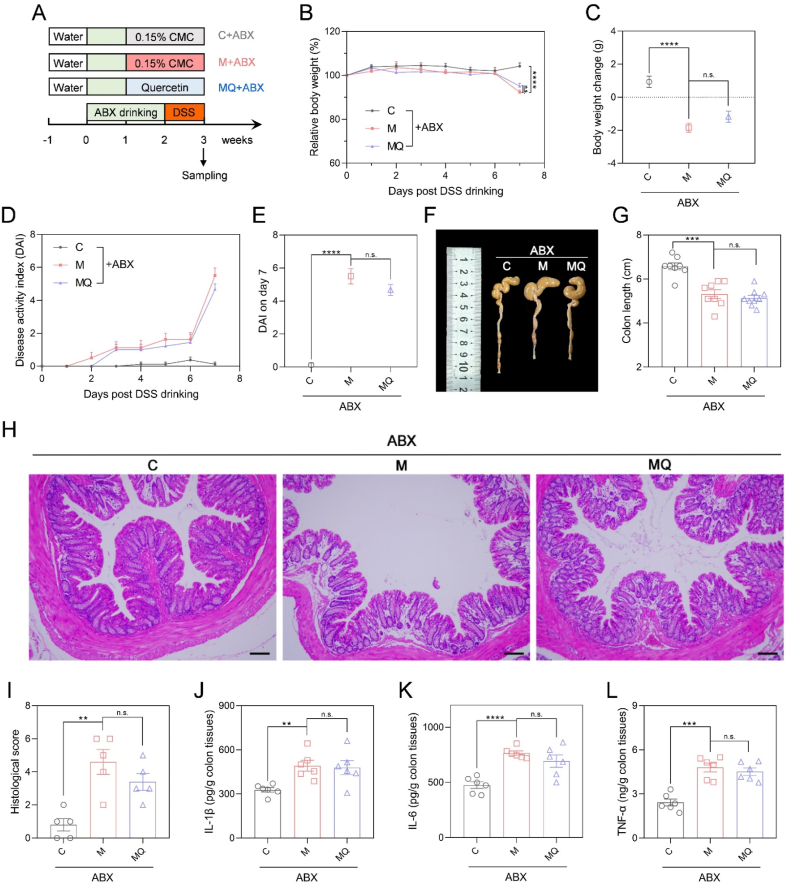
The ameliorative effects of quercetin on colitis was gut microbiota-dependent**.** (A) Study design of quercetin administration and colitis induction in microbiota-depleted mice with ABX drinking. (B) Relative body weight curve. (C) Body weight change in the DSS drinking period. (D, E) DAI curve and DAI on day 7 post DSS drinking. (F, G) Representative images of colon tissues and colon length statistics. (H, I) The H&E staining images of distal colon tissues and histological scores. Scale bar: 100 μm. (J–L) The colonic concentrations of IL-1β, IL-6, and TNF-α determined by ELISA. Data are shown as mean ± SEM, n = 8 per in B-G, n = 5 per group in I, n = 6 per group in J-L. The data between M + ABX and C + ABX or MQ + ABX groups was analyzed by one-way ANOVA with Tukey's post hoc test. ∗∗*p* < 0.01, ∗∗∗*p* < 0.001, ∗∗∗∗*p* < 0.0001, n.s., non-significant. Carboxymethylcellulose (CMC); antibiotics (ABX); dextran sulfate sodium (DSS); disease activity index (DAI); hematoxylin and eosin (H&E); interleukin 1β (IL-1β); interleukin 6 (IL-6); tumor necrosis factor α (TNF-α).

To further elucidate the role of gut microbes in quercetin's effects on colitis, we employed a gut microbiota-depleted murine model induced by ABX drinking (Fig. 4A). Compared to the mice without ABX treatment, more than 97 % of the gut bacterial taxa was eliminated in mice with ABX drinking (Fig. S11), verifying the microbial depletion efficiency. Notably, statistical analysis indicated that body weight decline, DAI, and colon length were similar between MQ and M groups following ABX treatment (Fig. 4B–G, S12), pointing to a major contribution of quercetin-induced gut microbiota reshape in the improvement of colitis. The colonic inflammation was then examined following the elimination of intestinal microbiota. It was found that ABX negated the substantial impact of quercetin in mitigating intestinal inflammation and reducing the spleen index in mice with colitis (Fig. 4H–L, S13, S14). The findings underscored that the remodeling of gut microbiota induced by quercetin was accountable for its positive effects on colitis.

### 3.5 Gut microbiota were essential for quercetin's protective effects on the intestinal barrier

**Fig. 5:**
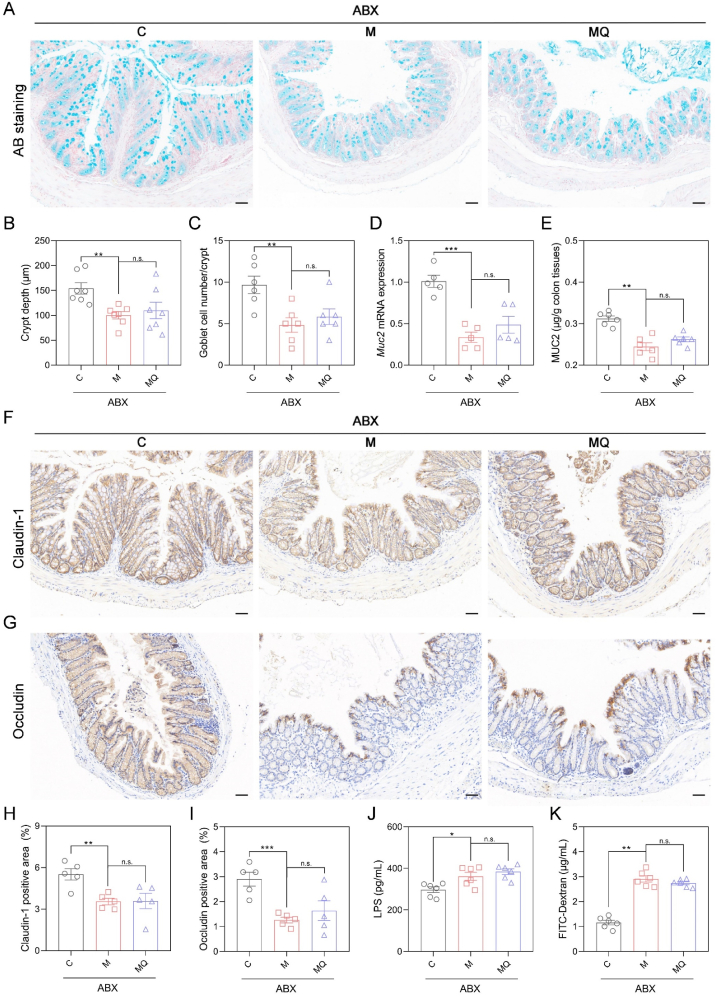
The protective effects of quercetin on intestinal barrier depended on gut microbiota. (A) AB staining images of distal colon tissues. Scale bar: 50 μm. (B) The crypt depth. (C) Goblet cell number per crypt. (D) The qRT-PCR results of colonic *Muc2* mRNA expression levels. (E) ELISA results of the colonic MUC2 content. (F, G) Representative immunohistochemical images of colonic sections stained with Claudin-1 and Occludin antibody. Scale bar: 50 μm. (H, I) The Claudin-1 and Occludin positive area statistics. (J) ELISA results of serum LPS concentration. (K) FITC-dextran concentrations in serum. Data are presented as mean ± SEM, n = 6 in the per group in B-E, J, and K, n = 5 per group in H and I. Comparisons between M + ABX and C + ABX or MQ + ABX groups were analyzed by one-way ANOVA with Tukey's post hoc test. ∗*p* < 0.05, ∗∗*p* < 0.01, ∗∗∗*p* < 0.001, n.s. non-significant. Alcian blue (AB); mucin-2 (Muc2); lipopolysaccharide (LPS); fluorescein isothiocyanate (FITC).

Subsequently, the protective role of quercetin against gut barrier damage were assessed in gut microbiota-depleted mice with colitis. AB staining and subsequent statistical analysis revealed no significant differences in crypt depth and goblet cell number between MQ and M groups following ABX treatment (Fig. 5A–C). Moreover, MUC2 expression remained similar between MQ and M groups after the removal of gut microbes (Fig. 5D and E). And the expression levels of Claudin-1 and Occludin did not exhibit any noticeable differences between MQ and M groups following ABX drinking (Fig. 5F–I and S15). The absence of significant variation in serum levels of LPS and FITC-Dextran between MQ and M groups further suggested that quercetin's protective effects on the gut barrier in colitis were diminished by microbiota depletion (Fig. 5J and K). These findings collectively indicated that gut microbiota were responsible for the enhanced gut barrier integrity induced by quercetin.

### 3.6 Quercetin enhanced the intestinal IVA generation in mice with colitis

**Fig. 6:**
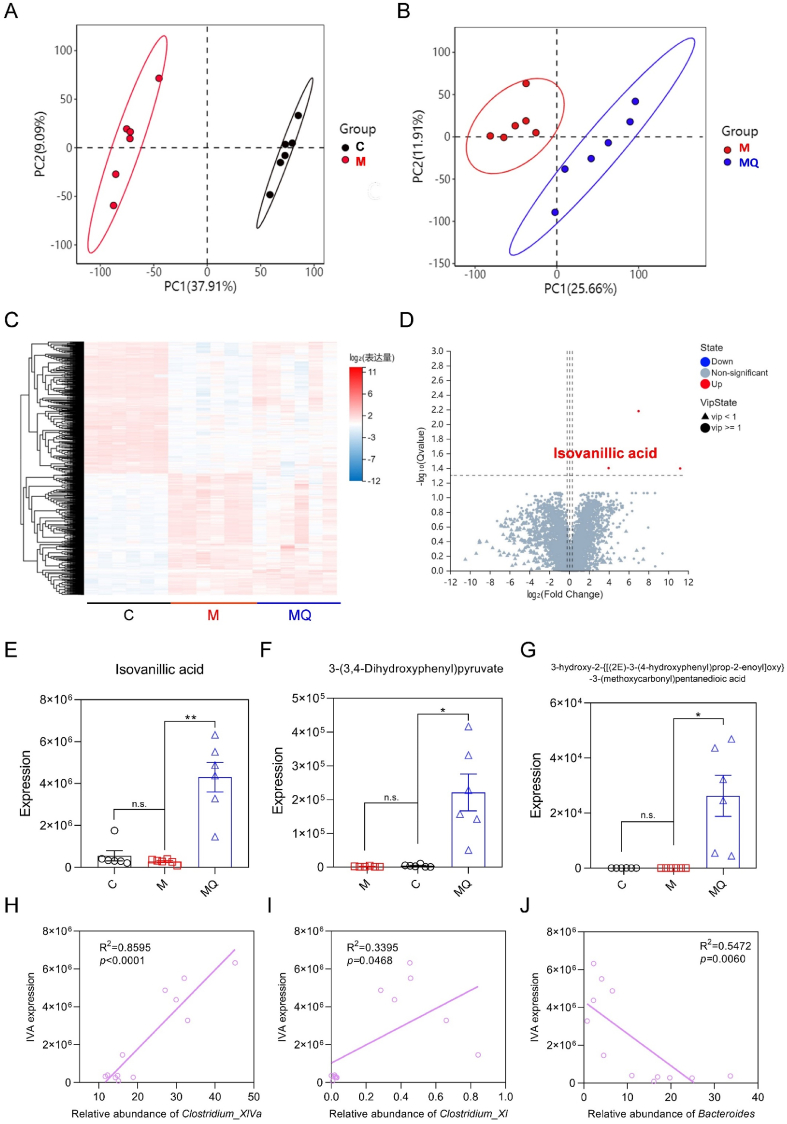
Quercetin enriched isovanillic acid (IVA) in mice with colitis**.** (A, B) Partial least squares discriminant analysis (PLS-DA) between C and M groups or M and MQ groups based on untargeted metabolomics. (C) Heatmap of differentially enriched metabolites in the feces. (D) Volcano plot of differentially enriched metabolites between M and MQ groups. (E–G) The relative expression levels of IVA, 3-(3,4-Dihydroxyphenyl) pyruvate, and 3-hydroxy-2-{[(2E)-3-(4-hydroxyphenyl) prop-2-enoyl]oxy}-3-(methoxycarbonyl) pentanedioic acid. (H–J) Linear regression analysis between fecal concentrations of IVA and relative abundance of *Clostridium_XIVa*, *Clostridium_XI*, and *Bacteroides*. Data are shown as mean ± SEM, n = 6 per group. The data between M and C or MQ groups was analyzed by one-way ANOVA with Tukey's post hoc test in E-G. ∗*p* < 0.05, ∗∗*p* < 0.01.

To better understand the main metabolites generated by gut microbiota that played a role in quercetin's anti-colitis effects, we conducted a non-targeted metabolomics analysis to examine differentially abundant metabolites in the feces of C, M, and MQ mice. The PLS-DA revealed substantial differences in the clustering patterns of fecal metabolites between M and C groups, as well as between MQ and M groups (Fig. 6A and B). These findings suggested that quercetin treatment significantly modified the fecal metabolite profile in mice with colitis. Next, heatmap and volcano plot analyses were performed on all metabolites identified through non-targeted metabolomics. The heatmap analysis revealed that quercetin partially ameliorated the dysregulation of fecal metabolites observed in mice with colitis (Fig. 6C). The volcano plot analysis highlighted the metabolites significantly modulated by quercetin treatment. It was demonstrated that quercetin significantly elevated the levels of IVA, 3-(3,4-Dihydroxyphenyl) pyruvate, and 3-hydroxy-2-{[(2E)-3-(4-hydroxyphenyl) prop-2-enoyl]oxy}-3-(methoxycarbonyl) pentanedioic acid in the feces of colitis-afflicted mice (Fig. 6D–G). Remarkably, quercetin treatment led to a 15-fold rise in IVA levels in the feces of mice with colitis (Fig. 6D). Furthermore, linear regression analysis demonstrated a notable positive link between IVA expression and the abundances of *Clostridium XIVa* and *Clostridium XI*, whereas a statistically significant inverse relationship was identified with the relative abundance of *Bacteroides* (Fig. 6H–J). These results collectively suggested that quercetin enhanced the concentration of intestinal IVA, potentially mediating its anti-colitis effects.

### 3.7 IVA markedly mitigated colitis induced by DSS

**Fig. 7:**
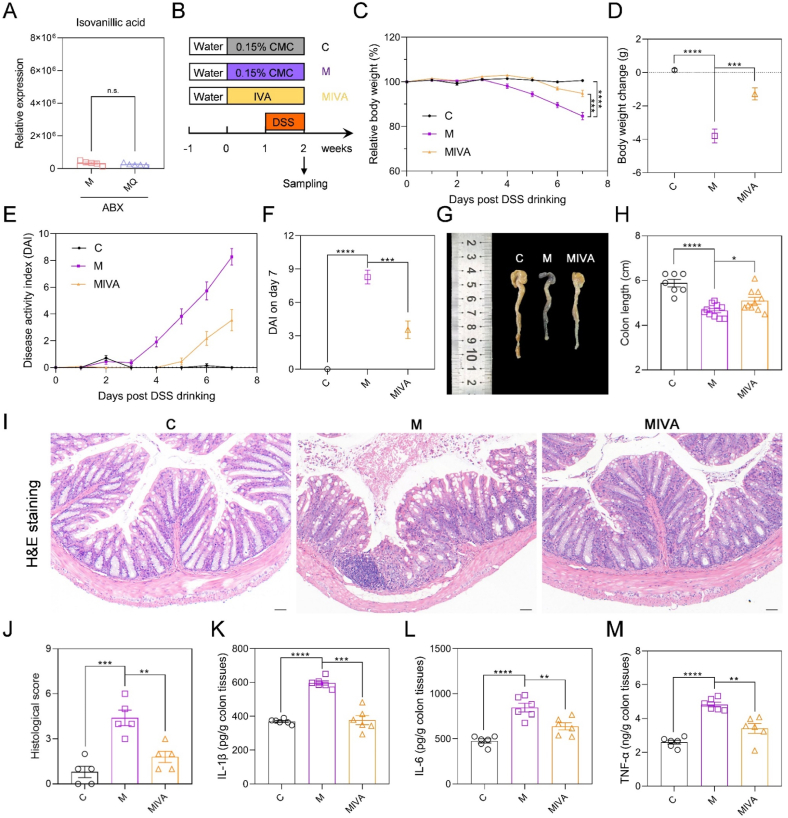
IVA significantly alleviated DSS-induced colitis. (A) The relative expression levels of IVA in gut microbiota-depleted mice with colitis. (B) Study design showing timeline of IVA administration and DSS drinking. (C) Relative body weight curve. (D) Body weight change in the DSS drinking period. (E, F) Disease activity index (DAI) curve and DAI on day 7 post DSS treatment. (G, H) Representative images of colon tissues and colon length. (I, J) The H&E staining images of distal colon tissues and histological scores. Scale bar: 100 μm. (K–M) The ELISA results of IL-1β, IL-6, and TNF-α concentrations in colon tissues. Data are shown as mean ± SEM, n = 7 in the control (C) group, n = 10 in the colitis model (M) group and IVA-administrated colitis model (MIVA) group in C-H, n = 5 per group in J, n = 6 per group in K-M. Comparisons between M + ABX and MQ + ABX groups were analyzed using a two-tailed Student's t-test in A, and differences between M and C or MIVA groups were analyzed by one-way ANOVA with Tukey's post hoc test in C-F, H, and J-M. ∗*p* < 0.05, ∗∗*p* < 0.01, ∗∗∗*p* < 0.001, ∗∗∗∗*p* < 0.0001. Carboxymethylcellulose (CMC); isovanillic acid (IVA); dextran sulfate sodium (DSS); disease activity index (DAI); hematoxylin and eosin (H&E); interleukin 1β (IL-1β); interleukin 6 (IL-6); tumor necrosis factor α (TNF-α).

To further substantiate the hypothesis that IVA was produced by gut microbiota, we quantified the fecal IVA content in both M and MQ mice following ABX treatment. Our findings revealed that ABX drinking markedly decreased the fecal concentration of IVA in the MQ group, restoring it to a level comparable to that observed in the M group (Fig. 7A). This evidence reinforced that the increase in intestinal IVA induced by quercetin was mediated by gut microbes.

To examine the potential anti-colitis effects of IVA, mice were orally administered 100 mg/kg of IVA following the same experimental protocol as utilized for quercetin treatment (Fig. 7B). It was found that IVA administration caused a significant improvement in DSS-induced weight reduction (Fig. 7C). On the 7th day of DSS drinking, the IVA-treated colitis mice experienced a 1.28 ± 1.19 g weight loss, compared to a weight drop of 3.80 ± 1.37 g in the M group (Fig. 7D). Further analysis of the DAI curve indicated that IVA markedly delayed the progression of colitis (Fig. 7E and S16), resulting in a 57.1 % reduction in the final DAI (Fig. 7F). Furthermore, the colonic shortening induced by DSS was significantly mitigated following the IVA administration (Fig. 7G and H). As evidenced by H&E staining, IVA significantly improved DSS-induced intestinal damage (Fig. 7I and J). More importantly, the observed reduction in the concentrations of intestinal inflammatory cytokines, along with the improvement in splenomegaly, in the MIVA group relative to the M group, underscored the inflammation-suppressive properties of IVA (Fig. 7K–M, S17, S18). Taken together, these findings confirmed that IVA from gut microbes was responsible for the anti-colitis properties of quercetin.

### 3.8 IVA ameliorated gut barrier damage in colitis

**Fig. 8:**
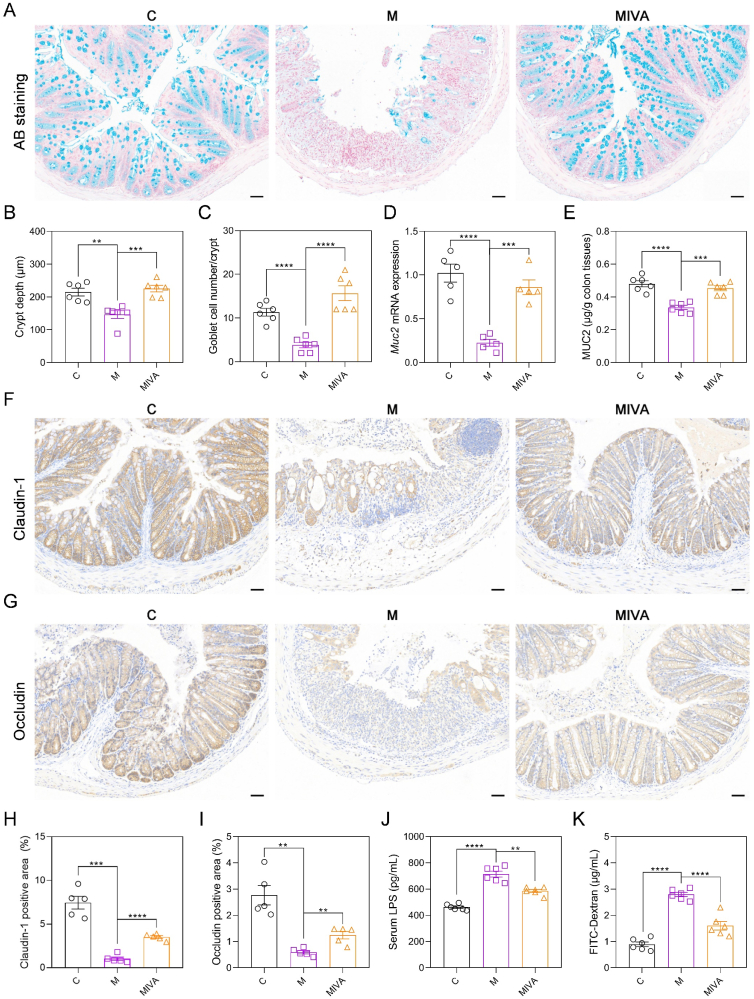
IVA enhanced intestinal integrity in colitis mice. (A) AB staining images of distal colon tissues. Scale bar: 50 μm. (B) The crypt depth. (C) Goblet cell number per crypt. (D) The qRT-PCR results of *Muc2* mRNA expression levels in colon tissues. (E) ELISA results of the colonic MUC2 content. (F, G) Representative immunohistochemical images of colonic sections stained with Claudin-1 and Occludin antibody. Scale bar: 50 μm. (H, I) The Claudin-1 and Occludin positive area statistics. (J) ELISA results of LPS concentration in serum. (K) FITC-dextran concentrations in serum. Data are presented as mean ± SEM, n = 6 in the per group in B-E, J, and K, n = 5 per group in H and I. Comparisons between M and C or MIVA groups were analyzed by one-way ANOVA with Tukey's post hoc test. ∗∗*p* < 0.01, ∗∗∗*p* < 0.001, ∗∗∗∗*p* < 0.0001. Alcian blue (AB); mucin-2 (Muc2); lipopolysaccharide (LPS); fluorescein isothiocyanate (FITC).

Next, the impact of IVA on the intestinal mucosal barrier was assessed through AB staining. AB staining and statistical results demonstrated that treatment with IVA ameliorated the reduction in crypt depth and the loss of goblet cells in mice with colitis (Fig. 8A–C). Subsequent qRT-PCR and ELISA assays indicated that IVA treatment counteracted the DSS-induced suppression of MUC2 expression (Fig. 8D and E). Furthermore, the higher colonic expression levels of Claudin-1 and Occludin of MIVA mice, as opposed to M mice, supported the beneficial impact of IVA on the gut barrier, as evidenced by immunohistochemical observations and analyses (Fig. 8F–I). This finding was further substantiated by qRT-PCR results, which demonstrated that IVA upregulated the mRNA expression levels of *Cldn-1* and *Ocln* in the colonic tissues of mice with colitis (Fig. S19). Serum LPS concentrations in the MIVA group were reduced by 18.0 % relative to the M group (Fig. 8J), implying that IVA contributed to the enhancement of intestinal integrity. Moreover, the results of the intestinal permeability test demonstrated that IVA significantly decreased FITC-Dextran levels in the serum of colitis mice by 42.7 % (Fig. 8K), thereby providing further evidence of IVA's protective role in gut barrier integrity. In summary, these observations suggested that IVA contributed positively to the repair of the intestinal barrier damage in murine models of colitis.

### 3.9 IVA alleviated LPS-induced intestinal barrier damage and inflammation *in vitro*

**Fig. 9:**
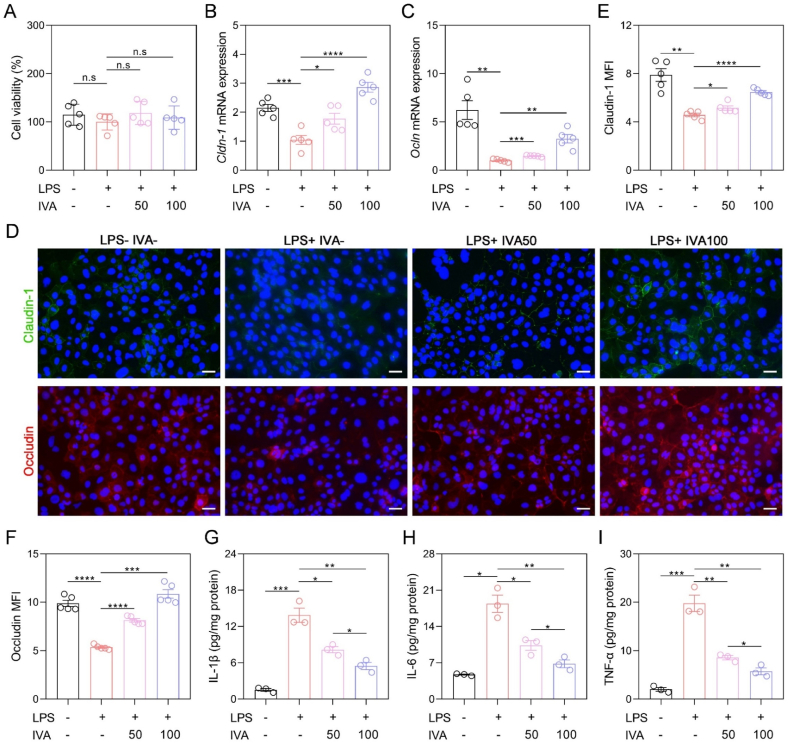
IVA alleviated LPS-induced epithelial barrier damage and inflammation *in vitro*. Caco-2 cells were treated with either 10 μg/mL LPS, 50 μM IVA, or 100 μM IVA, or none for 48 h. (A) Cell viability detected with CCK-8 kits. (B, C) The mRNA expression levels of *Cldn-1* and *Ocln* from qRT-PCR analysis. (D–F) Representative immunohistochemical images of colonic sections stained with Claudin-1 and Occludin antibody and statistics of MFI. Scale bar: 50 μm. (G–I) The ELISA results of IL-1β, IL-6, and TNF-α concentrations. Data are presented as mean ± SEM, n = 5 per group in A-F, n = 3 per group in G-I. The data was analyzed by one-way ANOVA with Tukey's post hoc test. ∗*p* < 0.05, ∗∗*p* < 0.01, ∗∗∗*p* < 0.001, ∗∗∗∗*p* < 0.0001. Lipopolysaccharide (LPS); isovanillic acid (IVA); interleukin 1β (IL-1β); interleukin 6 (IL-6); tumor necrosis factor α (TNF-α), mean fluorescence intensity (MFI).

So, did IVA exert a direct effect on intestinal epithelial cells to help restore the gut barrier? To verify this, we conducted an investigation into the effects of IVA on LPS-induced epithelial cell damage *in vitro*. The results indicated that co-incubation with 50 and 100 μM IVA for 48 h significantly upregulated the gene expression levels of *Cldn-1* and *Ocln* in LPS-challenged Caco-2 cells, while maintaining cell viability (Fig. 9A–C). Subsequent immunofluorescence staining and quantitative analyses demonstrated that LPS markedly decreased the expression levels of Claudin-1 and Occludin. However, co-incubation with IVA at concentrations of 50 and 100 μM significantly restored tight junction protein expressions (Fig. 9D–F). Furthermore, levels of pro-inflammatory cytokines were obviously declined in IVA + LPS-treated group compared to only LPS-treated group (Fig. 9G–I). These findings suggested that IVA was highly effective in mitigating inflammation and intestinal barrier damage triggered by LPS *in vitro*.

## 4 Discussion

UC has become a prominent public health issue due to its increasing incidence in both developed and developing countries (Kamm, 2017; Le Berre, Honap and Peyrin-Biroulet, 2023). The pathogenesis of UC involves multiple factors, including disrupted immune responses, intestinal microbiota imbalance, and gut barrier dysfunction (Chen et al., 2021; Kobayashi et al., 2020). Therefore, key treatment approaches focus on reestablishing intestinal microbiota homeostasis, controlling inflammation, and strengthening barrier function (Shi et al., 2024; Uchiyama et al., 2019). This study elucidated the protective roles and underlying mechanisms of quercetin against intestinal inflammation and barrier dysfunction. Our findings indicated that quercetin enhanced intestinal health by modulating the gut microbiota composition, notably enriching *Clostridium_XIVa* and *Clostridium_XI*, which in turn facilitated the generation of a key metabolite, IVA. The improvement in intestinal health was attributed to IVA's ability to protect the gut barrier and lessen inflammation. These results suggested that quercetin, along with IVA, might serve as a possible therapeutic option for UC and other gastrointestinal conditions, particularly those involving inflammation in future.

Quercetin, a prevalent natural flavonoid found in commonly consumed foods, serves dual purposes as a commercial dietary supplement and as a component in functional foods (Aghababaei and Hadidi, 2023; Ulusoy and Sanlier, 2020). Statistically, the daily human intake of quercetin through dietary sources varies between 10 and 500 mg (Michala and Pritsa, 2022). Importantly, even at daily doses reaching 2000 mg, quercetin has been reported to induce only mild or no symptoms of overdose in humans (Han et al., 2020). A prior daily dosage of 100 mg/kg body weight is referenced in this study (Wang et al., 2024). When converted from mouse doses to human equivalents, this dose translates to approximately 487.8 mg per day for an individual weighing 60 kg (Wojcikowski and Gobe, 2014). Quercetin's anti-inflammatory benefits have been documented in numerous studies, highlighting mechanisms such as activating the AMPKα1/SIRT1 pathway, inhibiting NLRP3 inflammasome and the NF-κB signaling pathway (Frenț et al., 2024). However, most orally ingested quercetin is not directly absorbed but rather influences the gut microbiome to exhibit probiotic-like effects (Frenț et al., 2024; Liu et al., 2025). Previous investigations into quercetin metabolism have demonstrated that quercetin and its hydrophilic conjugates, which remain unmetabolized in the small intestine, undergo biotransformations into various phenolic acids facilitated by gut bacteria (X. A. Zhu et al., 2024). The primary metabolites of quercetin produced by a healthy human microbiota include 3-hydroxyphenyl lactic acid, 4-hydroxybenzoic acid, 3,4-dihydroxybenzoic acid, and 2,4,6-trihydroxybenzoic acid (Di Pede et al., 2020). However, the gut-microbial metabolic pathways of quercetin in individuals with colitis remain inadequately understood, and the potential beneficial effects of its metabolites are yet to be fully elucidated. This investigation adopted an innovative strategy to delve into the mechanisms of quercetin by focusing on the gut flora and their metabolic processes. A notable discovery was that IVA, a phenolic acid generated by gut microbiota from quercetin, played a key role in mitigating colitis by strengthening gut barrier integrity and inhibiting the production of inflammatory cytokines. This finding substantially advanced our comprehension of how quercetin achieves its anti-inflammatory benefits through gut microbiota.

While the pathogenesis of UC remains unclear, evidence is accumulating that the dysbiosis of gut microbes is heavily implicated in the disease's development (Glassner et al., 2020; Ni et al., 2017). Sequencing analyses reveal notable variations in the gut microbiome in UC patients versus healthy individuals, primarily characterized by an increased number of conditionally pathogenic bacteria and a reduced presence of potentially probiotics (Wu et al., 2023). Thus, using dietary intervention to control the composition and function of gut microflora offers a novel approach for treating colitis. This study demonstrated that quercetin markedly lowered the relative level of *Bacteroides* in colitis-afflicted mice. Previous research has reported an increase in the proportion of these taxa in both UC animal models and patients (Mills et al., 2022; Zhang et al., 2024; Zhao et al., 2023). Although *Bacteroides* is generally commensal residents of the intestinal tract, they may turn pathogenic in the presence of gut dysbiosis and intestinal barrier damage (Zafar and Saier, 2021; Zhang et al., 2024). Moreover, we indicated that quercetin significantly elevated the intestinal content of *Butyricimonas*, a primary generator of short-chain fatty acids with anti-inflammatory properties, like butyrate (Jia et al., 2021), which might relate to the observed improvement in intestinal inflammation caused by quercetin. In contrast to prior researches, this study demonstrated a significant reduction in *Akkermansia* abundances following quercetin treatment (Liu et al., 2025). Although some studies have demonstrated the beneficial effects of *Akkermansia* on metabolic disorders and inflammation (Ioannou et al., 2025), others have shown that excessive proliferation of *Akkermansia* could disrupt the intestinal mucus barrier and thereby induce IBD (Khan et al., 2020; Wolter et al., 2024). Considering the dual impact of *Akkermansia* on the intestinal barrier, its involvement in colitis requires more exploration (He et al., 2023; Schwabkey et al., 2022). Notably, the *Clostridium XIVa* genus, which has been recognized as beneficial microorganisms in managing UC (Zhong et al., 2024), exhibited the highest enrichment following quercetin treatment. Furthermore, correlation analysis revealed a robust positive association between the *Clostridium XIVa* abundances and fecal IVA levels, suggesting their potential role as mediators in the conversion of quercetin to IVA. Certain *Clostridium* species, including *Clostridium perfringens* and *Clostridium orbiscindens*, have been identified as significant metabolizers of quercetin, leading to the generation of diverse phenolic acids (X. A. Zhu et al., 2024), which corroborated our findings. Further research is essential to elucidate how *Clostridium XIVa* transforms quercetin into IVA.

This study indicated that IVA, a gut microbiota-derived metabolite, was pivotal in facilitating the anti-colitis effects of quercetin. IVA (3-hydroxy-4-methoxybenzoic acid) is a natural phenolic acid occurring in various plant species, including Formosa koa (*Acacia confusa*), poonspar (*Calophyllum polyanthum*), and saffron (*Crocus sativus*) (Chen et al., 2023). IVA exhibits multiple bioactive properties, such as reducing inflammation, acting as an antioxidant, and lowering lipid levels (Chen et al., 2023). Nevertheless, research on the anti-colitis effects of IVA remains limited. Our findings suggested that IVA significantly ameliorated intestinal barrier dysfunction and inflammation in the colitis progression. It has been reported that IVA could mitigate lung inflammation induced by *Staphylococcus aureus* infection (Chen et al., 2023). Furthermore, VA, an isomer of IVA, has been documented to enhance gut barrier function in a compromised piglet model subjected to LPS challenge, and to attenuate UC in mouse models (Hu et al., 2022; Kim et al., 2010). Other phenolic acids, including caffeic acid (CA), protocatechuic acid (PA), syringic acid (SA), and gallic acid (GA), have demonstrated anti-colitis effects in different animal models (Lu and Han, 2024). Nevertheless, notwithstanding these promising outcomes, there remains an absence of thorough research and complete comprehension of how IVA and other phenolic acids work in treating UC.

While this study elucidated the role of the gut microbiota-IVA-intestinal barrier axis in the anticolitic effects of quercetin, there are several limitations. Firstly, the specific bacterial species responsible for the conversion of quercetin into IVA remain unknown, necessitating further investigation. Secondly, in-depth and systematic research is required on the pharmacology and toxicity of IVA in both animal and human studies. Thirdly, the anti-colitis effects of quercetin and IVA in human warrant further clinical investigation.

## 5 Conclusion

In conclusion, this investigation revealed that quercetin facilitated the production of IVA via modulating the gut microbiota composition, specifically by enriching *Clostridium_XIVa* and *Clostridium_XI*. This modulation resulted in the enhancement of gut barrier integrity and the inhibition of colonic inflammation, thereby mitigating DSS-induced colitis. Our study elucidated the critical involvement of the gut microbiota-IVA-intestinal barrier axis in the anticolitic effects of quercetin, thereby expanding the understanding of its pharmacological mechanisms in the treatment of UC. Additionally, our research underscored the potential of formulating nutritional supplements based on quercetin or IVA for the prevention of UC and other intestinal inflammatory disorders after further clinical validation.

## Author contributions

Liang Lei: Visualization, Methodology, Investigation, Funding acquisition. Wang Jing and Juangjuan Wang: Methodology, Investigation, Visualization, Writing – original draft. Wenjuan He, Tao Wu, Jing Li, and Xiaobin Bi: Investigation. Mei Mei and Xinlei Guan: Conceptualization, Methodology, Resources, Software, Validation, Writing – review & editing. Xiaoqiang Zhu: Funding acquisition, Conceptualization, Project administration, Resources, Visualization, Writing – review & editing.

## Funding

This study was funded by the National Natural Science Foundation of China (grant numbers 82304152, 32000282), Natural Science Foundation of Hubei Province (grant number 2023AFB392), Natural Science Foundation (Exploration Project) of Wuhan City (grant number 2024040801020373), the Funding for Scientific Research Projects from Wuhan Municipal Health Commission (grant numbers WX23Q28, WZ24B14, WX23Z68), Young Science and Technology Talents Morning Light Support Project of Hubei Province (grant number 202427) and Chenxing Project of Wuhan Municipal Health Commission.

## Declaration of competing interest

The authors declare that they have no known competing financial interests or personal relationships that could have appeared to influence the work reported in this paper.

## Data Availability

Data will be made available on request.
