# Supplementary material for: Quercetin boosts gut microbiota-driven production of isovanillic acid to alleviate colitis via enhancing intestinal barrier function
Source: Curr Res Food Sci. 2025 Aug 23;11:101183. doi: 10.1016/j.crfs.2025.101183 (PMC12405638; doi:10.1016/j.crfs.2025.101183)
Supplement: Multimedia component 1 [file mmc1.docx]

Supplementary Material

**Quercetin boosts gut microbiota-driven production of isovanillic acid to alleviate colitis via enhancing intestinal barrier function**

**Liang Lei**^1,2#^**, Jing Wang**^3#^**, Juanjuan Wang**^3#^**, Wenjuan He**^1,2^**, Tao Wu**^1,2^**, Jing Li**^4^**, Xiaobin Bi**^5^**, Mei Mei**^6*^**, Xinlei Guan**^1,2*^**, Xiaoqiang Zhu**^1,2^^*^

^1^ Central Laboratory, Wuhan Fourth Hospital, Wuhan, China

^2^ Institute of Clinical Pharmacy, Wuhan Fourth Hospital, Wuhan, China

^3^ Department of Gastroenterology, Wuhan Fourth Hospital, Wuhan, China

^4^ Pharmaceutical Department, Hubei Cancer Hospital, Tongji Medical College, Huazhong University of Science and Technology, Wuhan, China

^5^ Hubei Key Laboratory of Natural Medicinal Chemistry and Resource Evaluation, School of Pharmacy, Tongji Medical College, Huazhong University of Science and Technology, Wuhan, China.

^6^ Cancer Center, Union Hospital, Tongji Medical College, Huazhong University of Science and Technology, Wuhan, China.

^#^ Liang Lei, Jing Wang, and Juanjuan Wang contributed equally to this work.

^*^ Correspondence to: Mei Mei, mei_mei@hust.edu.cn; Xinlei Guan, xinleiguan@hust.edu.cn; Xiaoqiang Zhu, zhuxiaoqiang1992@126.com.


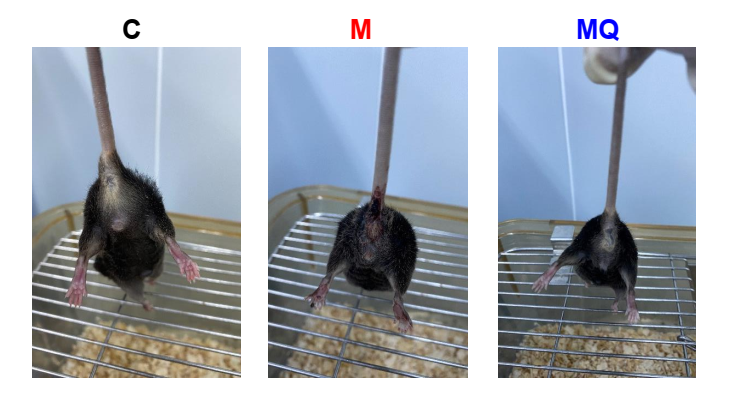


Figure S1. Representative images of mice from C, M, and MQ groups.


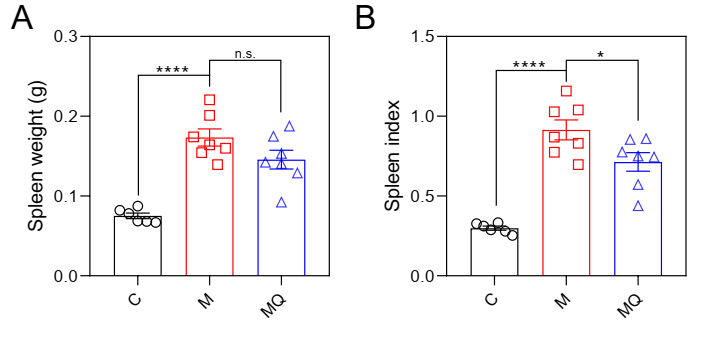


Figure S2. The spleen weight (A) and spleen index (B) of C, M, and MQ groups. Data are presented as mean ± SEM, n=6 in the C group, n=7 in M and MQ groups, **p* < 0.05, *****p* < 0.0001, n.s., non-significant.


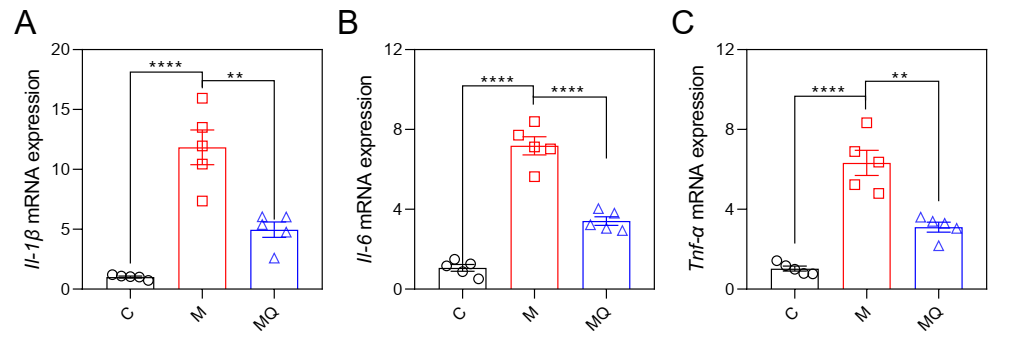


Figure S3. The mRNA expression levels of *Il-1β* (A)，*Il-6* (B), and *Tnf-α* (C) in colon tissues. Data are shown as mean ± SEM, n=5 in each group, ***p* < 0.01, *****p* < 0.0001.


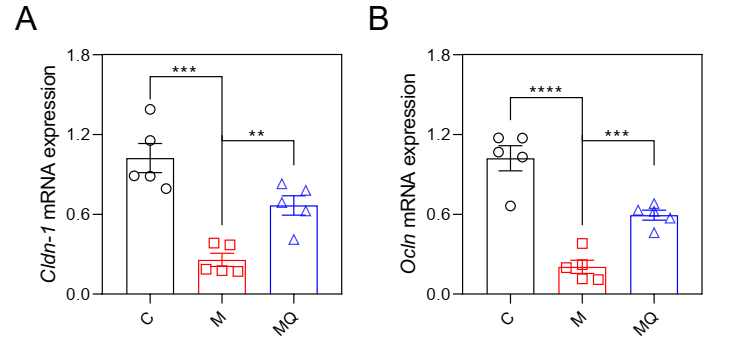


Figure S4. The mRNA expression levels of *Cldn-1* (A) and *Ocln* (B) in colon tissues. Data are shown as mean ± SEM, n=5 in each group, ***p* < 0.01, ****p* < 0.001, *****p* < 0.0001.


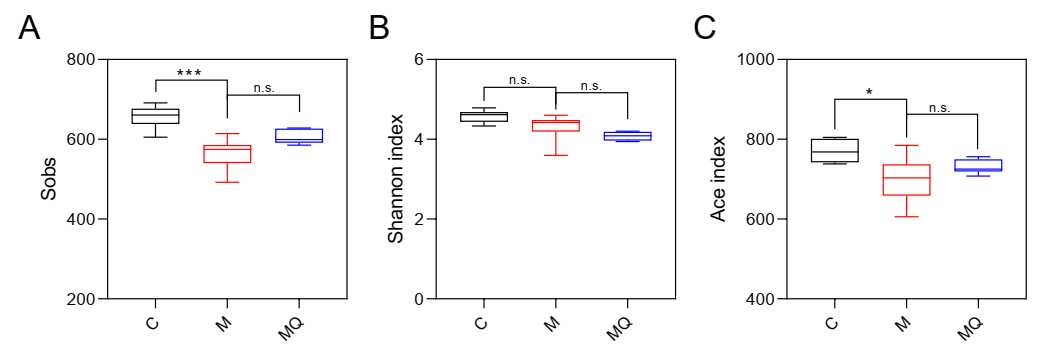


Figure S5. The α diversity analysis of gut microbiota. (A) Sobs. (B) Shannon index. (C) Ace index. Data are shown as interquartile range, n=6 in each group, **p* < 0.05, ****p* < 0.001, n.s., non-significant.


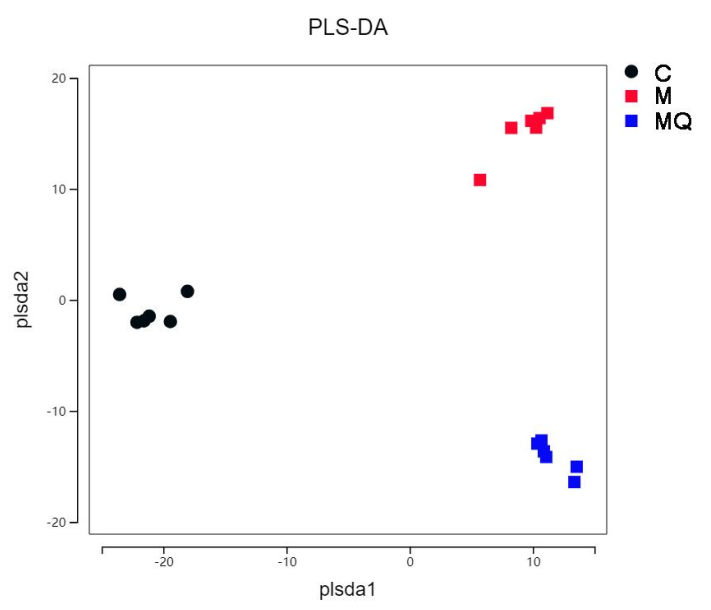


Figure S6. Partial least squares discriminant analysis (PLS-DA) among C, M, and MQ groups based 16S rDNA sequencing results, n=6 in each group.


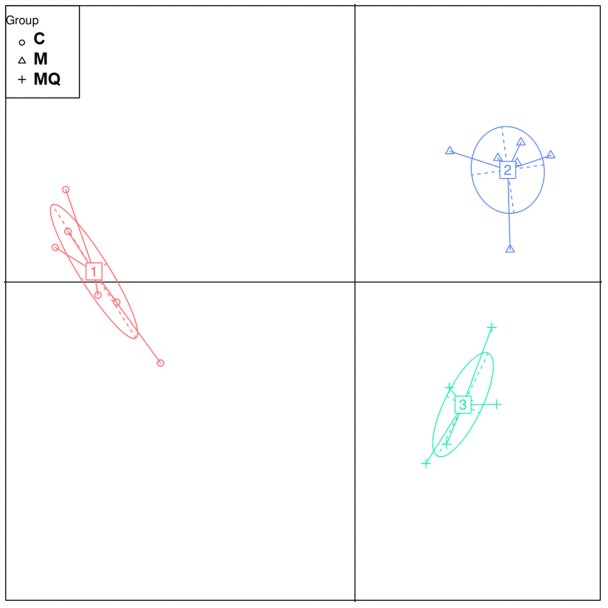


Figure S7. Enterotype analysis among C, M, and MQ groups, n=6 in each group.


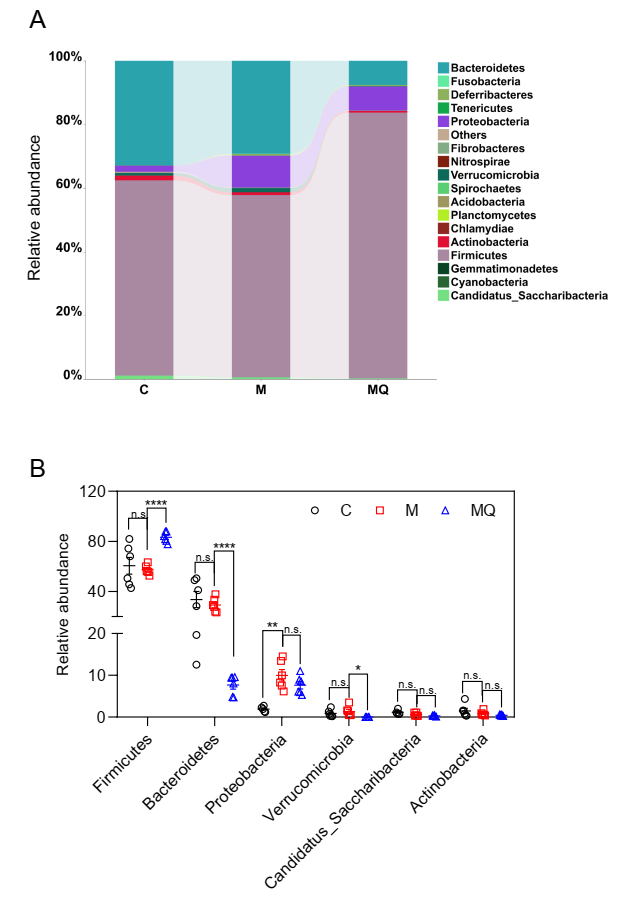


Figure S8. The gut microbiota analysis at the phylum level. (A) Microbiota structure at the phylum level. (B) Relative abundances of Firmicutes, Bacteroidetes, Proteobacteria, Verrucomicrobia, Candidatus_Saccharibacteria, and Actinobacteria. Data are presented as mean ± SEM, n=6 in each group, **p* < 0.05, ***p* < 0.01, *****p* < 0.0001, n.s., non-significant.


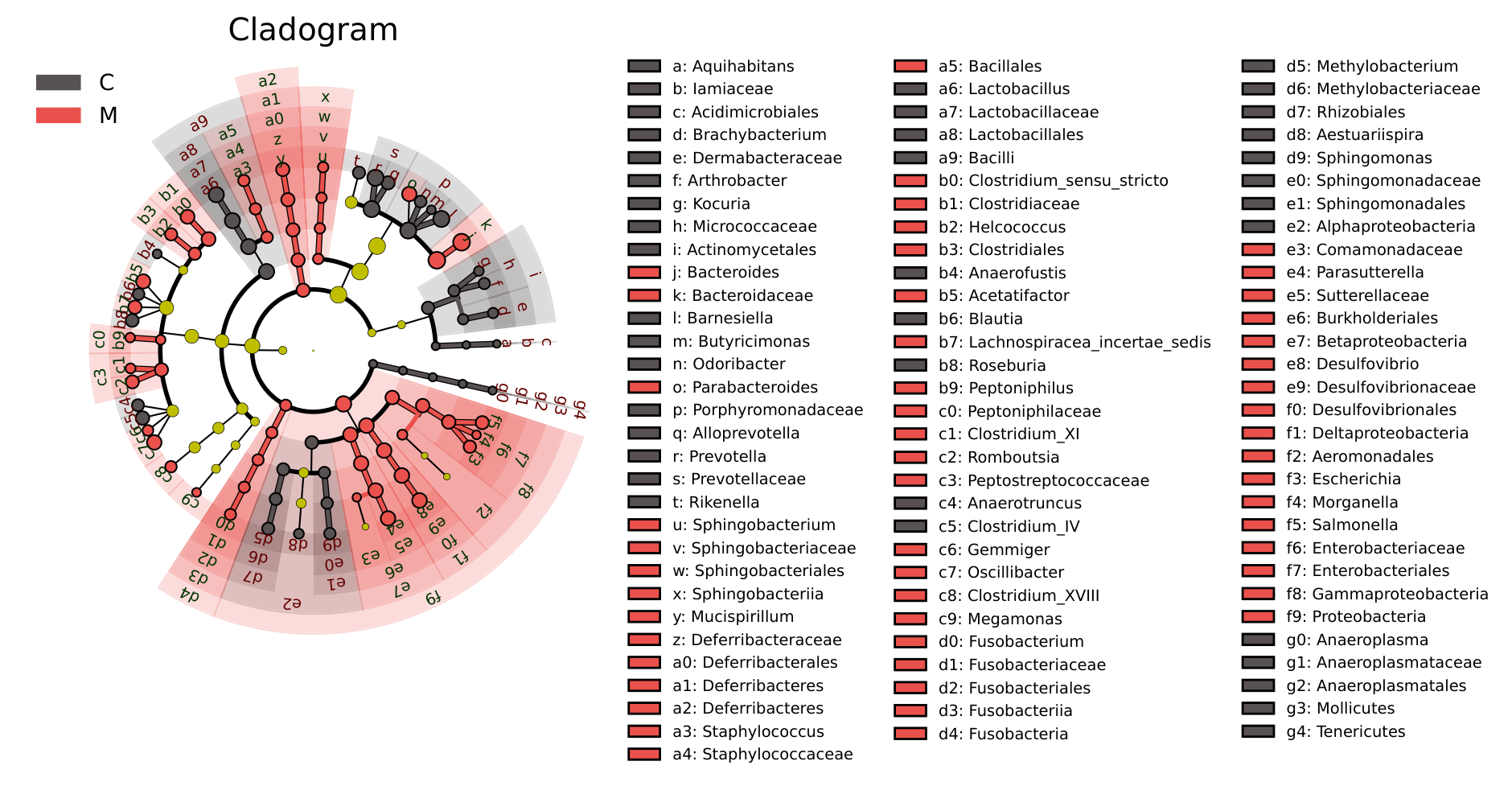


Figure S9. The linear discriminant analysis of effect size (LEfSe) of gut microbiota between C and M groups.


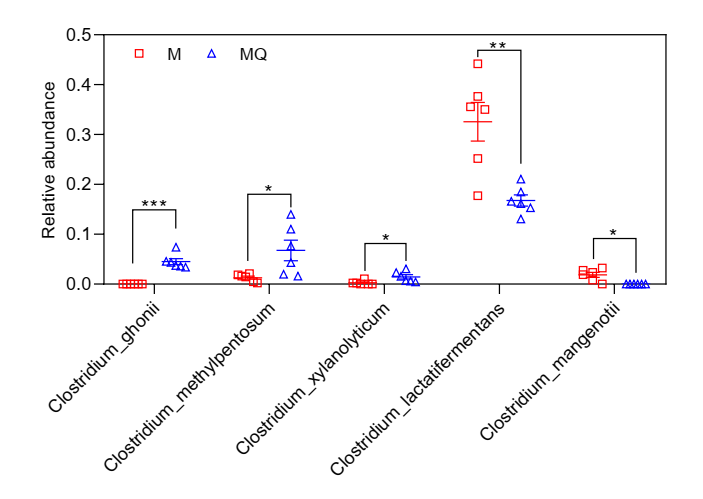


Figure S10. Relative abundances of *Clostridium_ghonii*, *Clostridium_methylpentosum*, *Clostridium_xylanolyticum*, *Clostridium_lactatifermentans*, and *Clostridium_mangenotii*. Data are presented as mean ± SEM, n=6 in each group, **p* < 0.05, ***p* < 0.01, ****p* < 0.001.


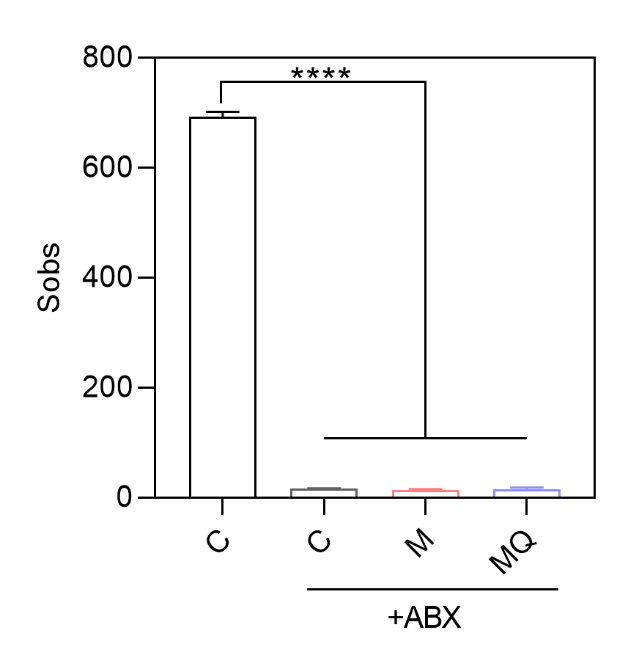


Figure S11. Sobs statistics showing microbial depletion efficiency with ABX drinking, n=4 in each group, *****p* < 0.0001.


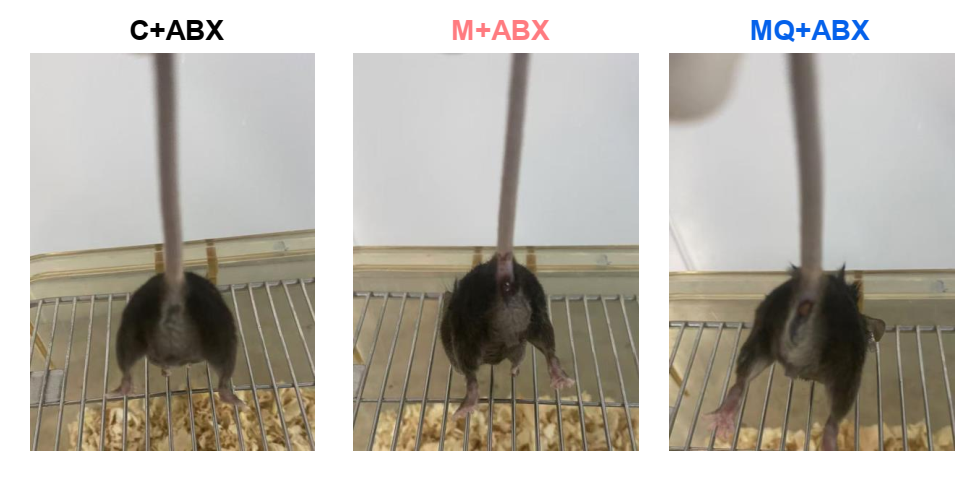


Figure S12. Representative images of ABX-drinking mice from C, M, and MQ groups.


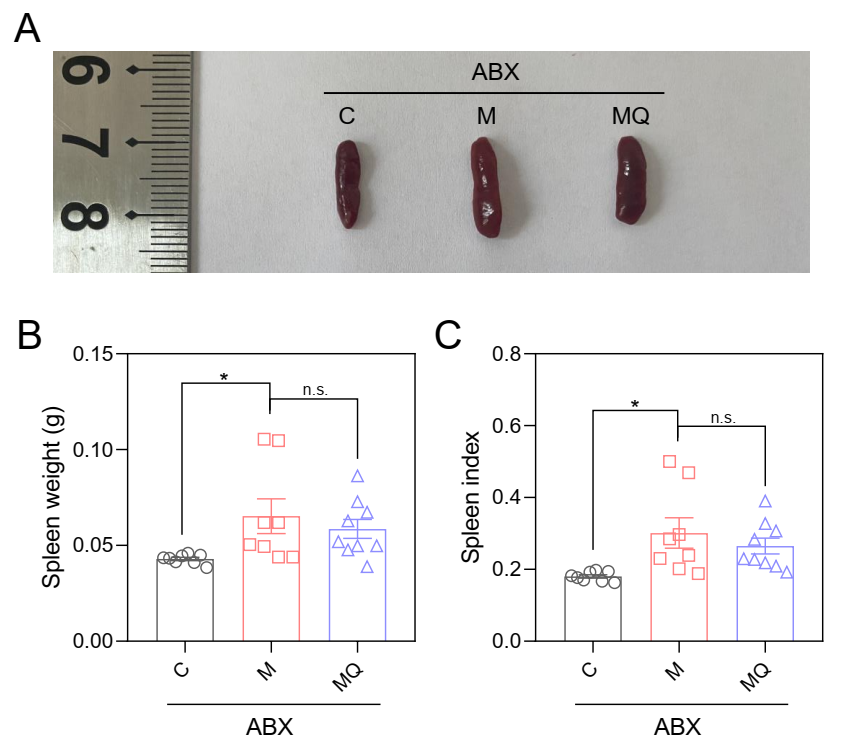


Figure S13. Representative images of spleen (A), the spleen weight (B), and spleen index (C) of mice from C+ABX, M+ABX, and MQ+ABX groups. Data are presented as mean ± SEM, n=8 in each group, **p* < 0.05, n.s., non-significant.


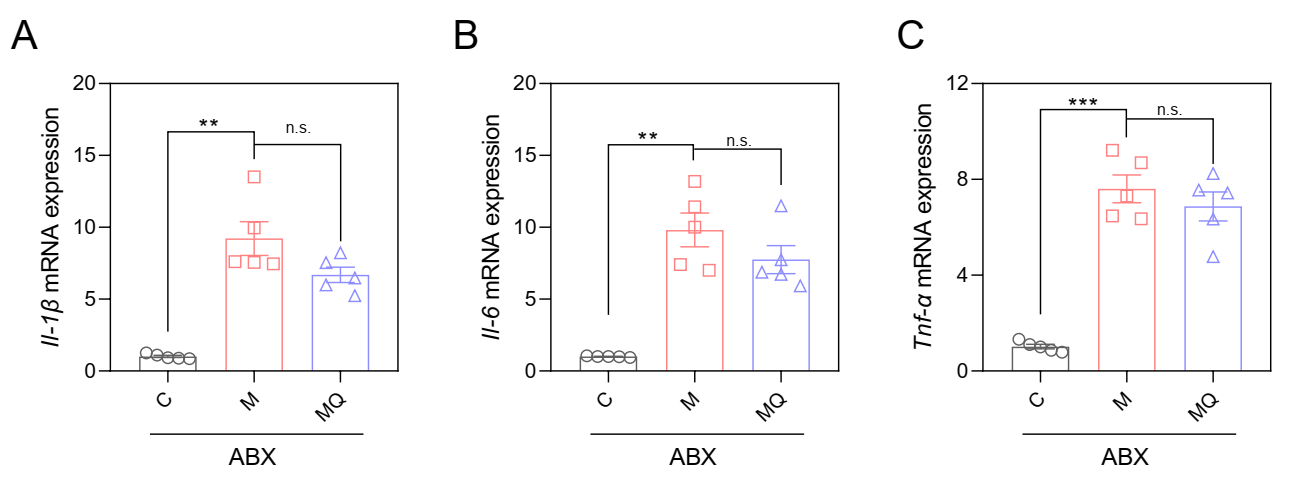


Figure S14. The mRNA expression levels of *Il-1β* (A)，*Il-6* (B), and *Tnf-α* (C) in colon tissues from gut microbiota-depleted mice. Data are shown as mean ± SEM, n=5 in each group, ***p* < 0.01, ****p* < 0.001, n.s., non-significant.


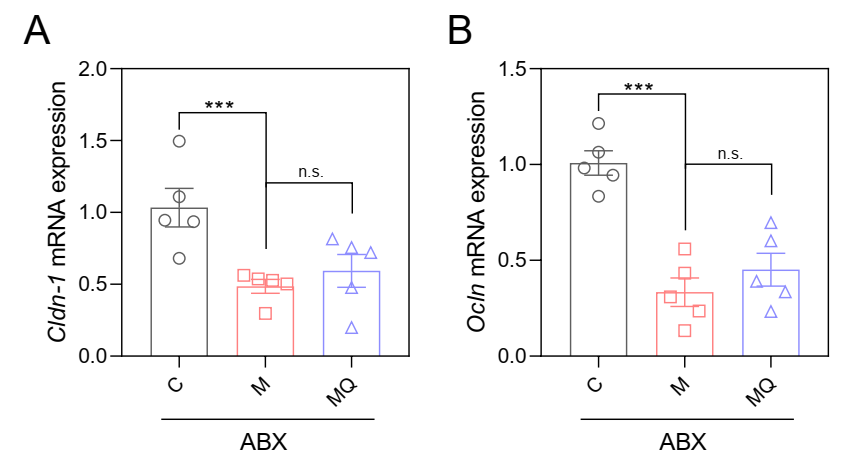


Figure S15. The mRNA expression levels of *Cldn-1* (A) and *Ocln* (B) in colon tissues from gut microbiota-depleted mice. Data are shown as mean ± SEM, n=5 in each group, ****p* < 0.001, n.s., non-significant.


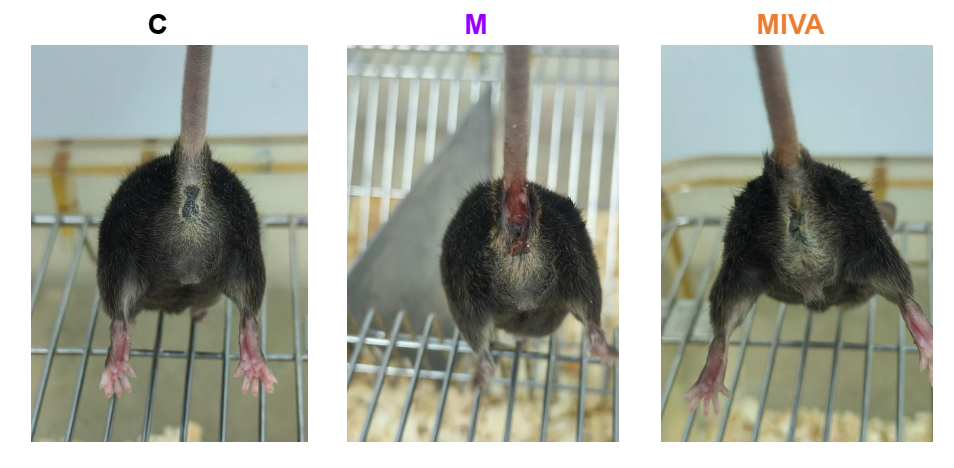


Figure S16. Representative images of mice from C, M, and MIVA groups.


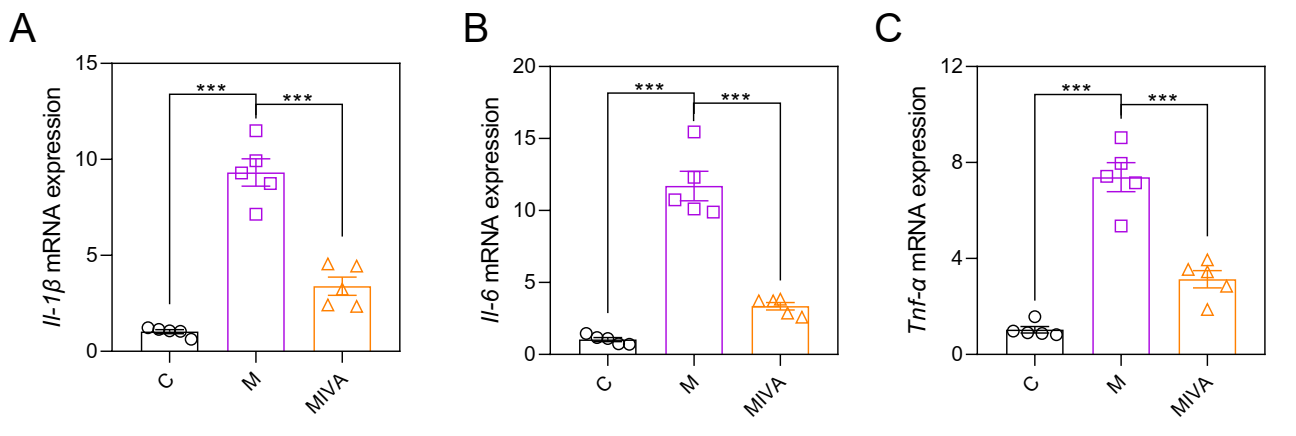


Figure S17. The mRNA expression levels of *Il-1β* (A)，*Il-6* (B), and *Tnf-α* (C) in colon tissues. Data are shown as mean ± SEM, n=5 in each group, ****p* < 0.001.


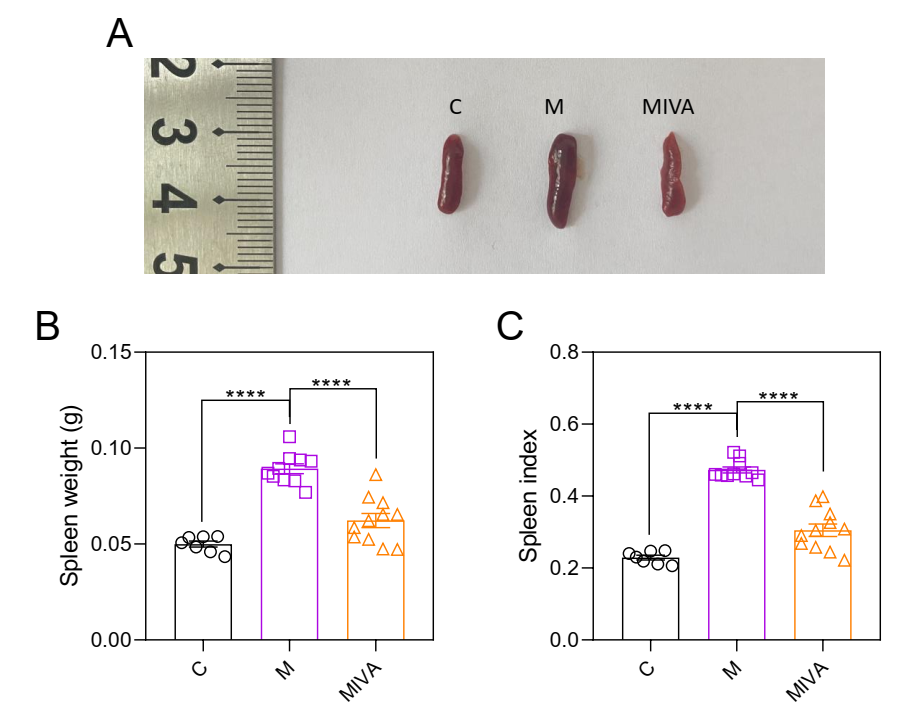


Figure S18. Representative images of spleen (A), the spleen weight (B), and spleen index (C) of mice from C, M, and MIVA groups. Data are shown as mean ± SEM, n=7 in the C group, n=10 in the M and MIVA group, *****p* < 0.0001.


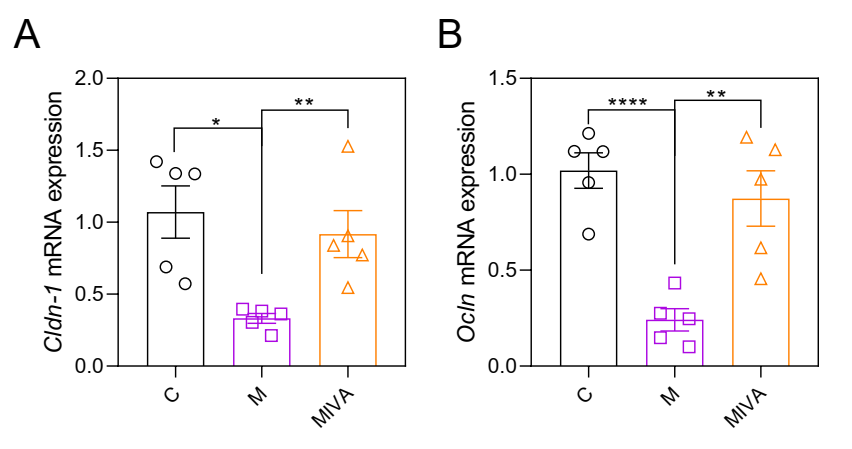


Figure S19. The mRNA expression levels of *Cldn-1* (A) and *Ocln* (B) in colon tissues. Data are shown as mean ± SEM, n=5 in each group, **p* < 0.05, ***p* < 0.01, *****p* < 0.0001.

**Table S1. Primers used for quantitative real-time PCR**

|  | **Gene** | **Forward primer (5’-3’)** | **Reverse primer (5’-3’)** |
| --- | --- | --- | --- |
| *Mouse* | *Gapdh* | GTTCCTACCCCCAATGTGTCC | TAGCCCAAGATGCCCTTCAGT |
|  | *Il-1β* | AGCCGAGGTCTGCATTACATT | TGGCAGTCTGGATAACTGATGA |
|  | *Il-6*  *Tnf-α* | CCTGAGACTCAAGCAGAAATGG  ATGTCGGCTCCAGGACCTTA | AGAAGGAAGGTCGGCTTCAGT  GGTAGTAACTGTTGACACCCACT |
|  | *Muc2* | ATGCCCACCTCCTCAAAGAC | GTAGTTTCCGTTGGAACAGTGAA |
|  | *Cldn-1*  *Ocln* | CCTATGACCCCAGTCAATGC  AGGAACCGAGAGCCAGGT | ACAGCAAAGTAGGGCACCTC  TGAGCAATGCCCTTTAGCTT |
| *Human* | *Gapdh* | GGAGCGAGATCCCTCCAAAAT | GGCTGTTGTCATACTTCTCATGG |
|  | *Cldn-1* | CCTCCTGGGAGTGATAGCAAT | GGCAACTAAAATAGCCAGACCT |
|  | *Ocln* | ACAAGCGGTTTTATCCAGAGTC | GTCATCCACAGGCGAAGTTAAT |
